# Analysis of long-term statistical data of cobalt flows in the EU

**DOI:** 10.1016/j.resconrec.2021.105690

**Published:** 2021-10

**Authors:** María Fernanda Godoy León, Gian Andrea Blengini, Jo Dewulf

**Affiliations:** a Research Group Sustainable Systems Engineering (STEN), Ghent University, Coupure Links 653, Ghent 9000, Belgium; b European Commission, Joint Research Centre (JRC), Via E. Fermi 2749, Ispra, VA 21027, Italy

**Keywords:** Cobalt, Critical raw materials, Long-term statistical data, International trade, Domestic production

## Abstract

Long-term statistical data was explored, acquired, processed, and analysed in order to assess the historical domestic production and international trade of a number of cobalt-containing commodities in the EU. Different data sources were examined for data, such as the British Geological Survey (BGS), the US Geological Survey (USGS), and the Eurostat and UN Comtrade (UNC) databases, considering all EU-member states before and after they joined the EU. For the international trade, hidden flows related to data gaps such as data reported in monetary value or recorded as “special category” were identified and included in the analysis. In addition, data from the Finnish customs database (ULJAS) was used to complement flows reported by Eurostat and UNC. From UNC, data was obtained considering the member states as reporters or as partners of the trade, due to internal differences of the database.

Based on the acquired data the domestic production and international trade of the commodities were reconstructed for the timeframes 1938–2018 and 1988–2018, respectively. Next to the analysis of the trend of the production and trade of the different commodities, the importance of including hidden flows was revealed, where hidden flows represented more than 50% of the flow of a year in some cases. In addition, it was identified that even from reliable data sources, strong differences (more than 100% in some cases) can be found in the reported data, which is crucial to consider when utilizing the data in research.

## Graphical abstract

**Figure:**
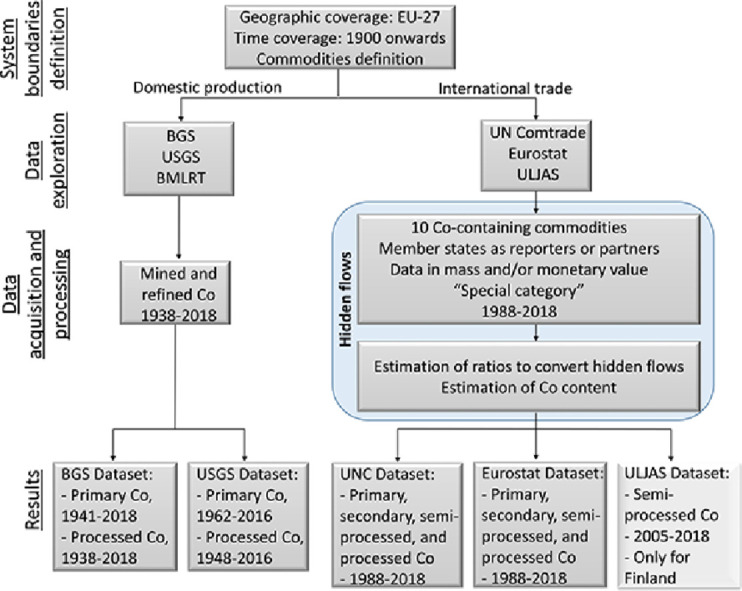

## 1 Introduction

Cobalt (Co) is a critical/strategic material for a number of economies (e.g. Japan, South Korea, USA, Australia, EU) (Hatayama and Tahara, 2015; USGS, 2018; Commonwealth of Australia, 2019; European Commission, 2020a; Lee and Cha, 2021). In the EU, the metal has been classified as such since 2011, due to its economic importance and its vulnerability to supply disruption (European Commission, 2020a). Cobalt is used in applications in industry, transport, and households, as in hard metals, magnets, superalloys, and batteries; being the latter key in the transition from fossil fuel to more sustainable energy sources. In addition, the EU depends strongly on imports of the metal, e.g. in 2016 its domestic production of primary material only covered around 7% of the consumer's demand (Matos et al., 2020).

Due to the importance of the metal, the Co cycle has been studied globally and regionally, fully or partially addressing its supply, demand, stocks and flows. Many of these studies have been developed through Material Flow Analysis (MFA) tools, applying both static and dynamic models (National Research Council, 1983; Shedd, 1993; Harper et al., 2012; BIO by Deloitte, 2015; Zeng and Li, 2015; Sun et al., 2019; Matos et al., 2020; Godoy León and Dewulf, 2020). For the EU, a number of studies have been carried out. Some of them have targeted inventory data (data to establish an MFA, e.g., value of parameters, flows) (RPA, 2012; Godoy León and Dewulf, 2020), and many others have focused on scenarios for future demand and prospective dynamic MFA (dMFA) (Ait Abderrahim and Monnet, 2018; Alves Dias et al., 2018; Deetman et al., 2018; Bobba et al., 2019; Tercero, 2019; Godoy León et al., 2020). However, little has been done to study the historical stocks and flows of Co in long-term retrospective analyses. Studies, such as Zeng and Li (2015) and Sun et al. (2019) have used retrospective dMFA to trace the Co stocks and flows in China between 2005 and 2013, and on a global scale between 1995 and 2015, respectively. For other metals such as copper, there are long-term retrospective analyses at the EU level, e.g. Soulier et al. (2018) and Ciacci et al. (2017), although that is not the case for Co. To the best of the authors’ knowledge, at the EU level only the Co demand for the EU has been estimated for the period 2000–2017 (Tercero, 2019). Static MFA studies have been developed for the year 2012 and the period 2012–2016 (BIO by Deloitte, 2015; Matos et al., 2020; Godoy León et al., 2021) although these did not go further on the analysis about how the anthropogenic stock has built up in the EU. The latter also identified hidden flows in the Co value chain; the flow of cobalt intermediate commodities originally acquired from the Eurostat database presented gaps that were filled using data from sources such as the UN Comtrade database and the Finnish customs database. The study did not dig deeper in this matter, but it set as a precedent to be taken into account in further studies.

Considering the importance of Co for the EU, a proper understanding of its historical metabolism in the region is crucial. It is needed to understand shifts and trends in cobalt flows, and to identify potential sources of secondary material originating from earlier stock build-up, which are crucial to enhance circularity in the economy. Furthermore, it is also required to improve forecasting assessments that are key in the discussion of the management of the metal. Open data sources, such as geological surveys, compile data about the historical domestic production and international trade of a number of commodities related to Co. This data has been used in MFA studies such as BIO by Deloitte (2015) and Matos et al. (2020) analysing limited periods; however, to the best of the authors’ knowledge, it has never been analysed as a whole by comparing different long-term datasets, evaluating data gaps and hidden flows in the Co value chain, and it has not been used to assess the historical metabolism of Co in the EU over a long-term period.

In this line, the objective of this work is to explore different data sources in a long-term perspective, to analyze the domestic production and international trade of a number of Co-containing commodities, identifying and filling data gaps. The focus is on current EU-member states (the analysis of historical trade routes is out of the scope). The exploration of data was set from 1900 onwards. The final goal is to reconstruct the flows of Co in the EU over the last decades, to be used in a retrospective dMFA in a following step.

## 2 Methodology

**Fig. 1:**
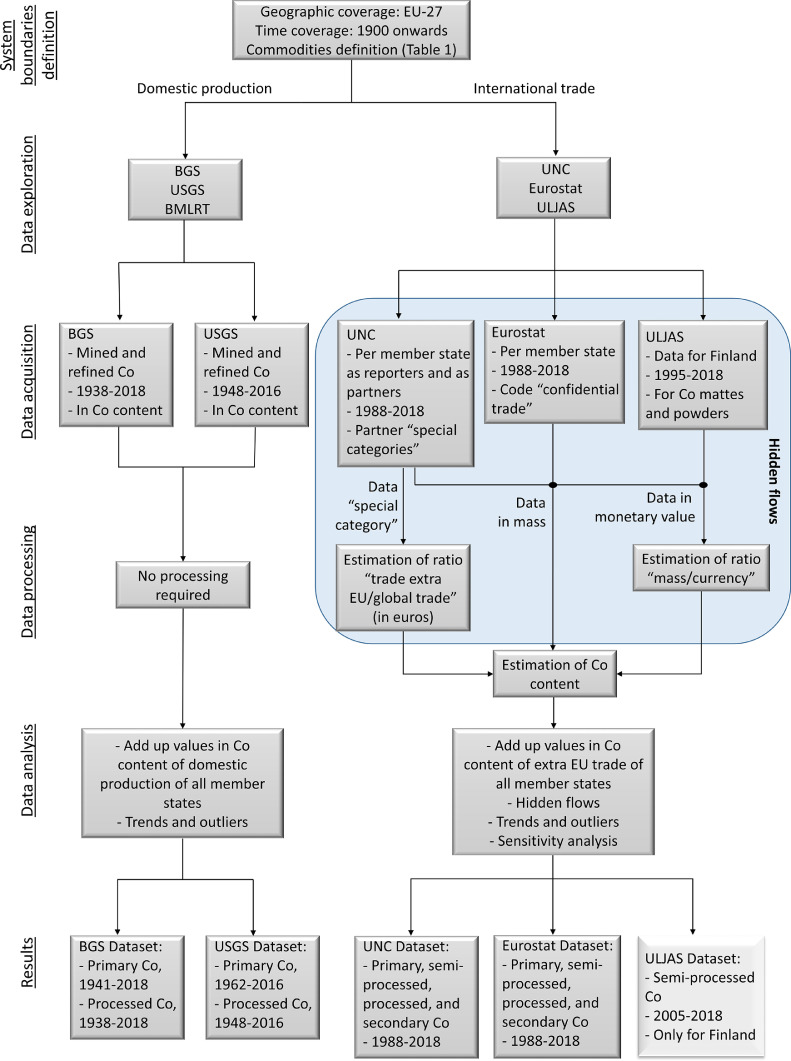
Schematic summary of the applied methodology. BGS: British Geological Survey, USGS: US Geological Survey, BMLRT: Austrian Federal Ministry of Agriculture, Regions and Tourism, UNC: UN Comtrade, ULJAS: Finnish customs database. The ULJAS dataset is shown in a different color, as its purpose is to fill the gaps of the datasets from UNC and Eurostat.

This section is divided in two main parts. First, the system boundaries are defined. Then, the methodology for the exploration, acquisition, processing, and analysis of long-term statistical data is described. Fig. 1 presents a summary of the used methodology.

### 2.1 System boundaries

Long-term historical production and trade of Co were studied for the EU-27 through different phases of its lifecycle, from mining to manufacturing, considering also the recycling of waste and scrap. The study was done considering the 27 member states that are currently part of the EU, even before they joined the union. In the same line, the United Kingdom (UK) was not included in the study, since it left the EU in 2020 (European Commission, 2020b). This selection of member states allows for the analysis of the historical flows of Co for the current EU, which is required for a better understanding of the present and future societal metabolism of Co in the region.

**Fig. 2:**
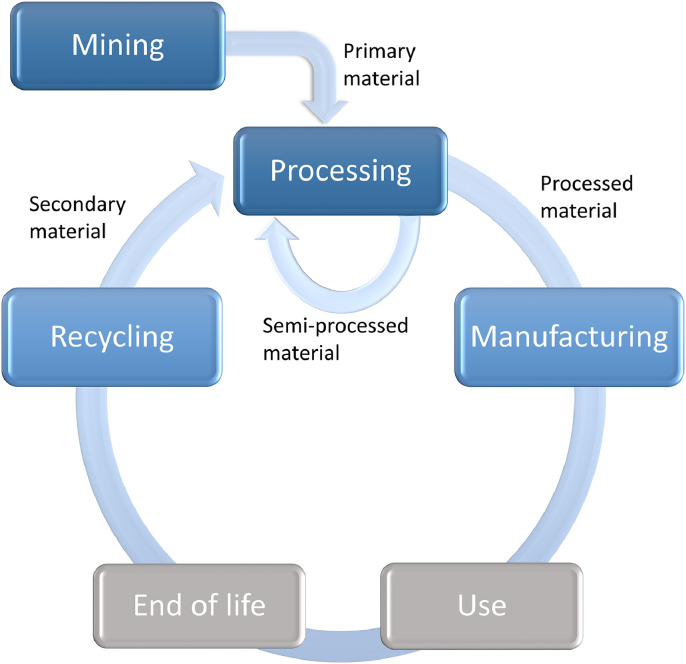
Simplified lifecycle of Co. Commodities related to the gray rectangles are out of the scope. The recycling phase also considers recycling of waste and scrap coming from the processing and manufacturing phases.

A number of Co-containing commodities were defined based on the PRODCOM code and the CN (combined nomenclature) code, following the methodology of Matos et al. (2020). The commodities were categorised as primary, secondary, semi-processed, and processed material, according to their location in the lifecycle of the metal (see Table 1 and Fig. 2). Table 1 lists the analysed commodities per category, linking their PRODCOM and CN code, and the explored sources. The timeframe for the data exploration (i.e., assessment of data availability) was established from 1900 onwards.

**Table 1:** List of codes for Co-containing commodities considered in the study. PRC: PRODCOM code, CN: Combined Nomenclature code. BGS: British Geological Survey, USGS: US Geological Survey, BMLRT: Austrian Federal Ministry of Agriculture, Regions and Tourism, UNC: UN Comtrade, ULJAS: Finnish customs database. * From this commodity, the amounts of Co mattes and intermediates and of Co powders were estimated. In addition, it is assumed that this commodity includes crude Co hydroxides.

Category	Commodity	PRC 2019	CN 2019	Explored source
Primary	Mineral cobalt	–	–	BGS, USGS, BMLRT
Primary/ Semi-processed	Cobalt ores and concentrates	7291905	2605 00 00	Eurostat, UNC
Semi-processed/ Processed	Cobalt mattes and other intermediate products of cobalt metallurgy; unwrought cobalt; cobalt powders*	24453035	8105 10 10	Eurostat, UNC, ULJAS
			8105 20 00	
Semi-processed	Nickel mattes	24451210	7501 10 00	Eurostat, UNC
Secondary	Waste and scrap, of nickel alloys	–	7503 00 90	Eurostat, UNC
Secondary	Cobalt waste and scrap (excl. ash and residues containing cobalt)	–	8105 30 00	Eurostat, UNC
Secondary	Ash and residues containing mainly cobalt	26209080	–	Eurostat
		26209970		
Processed	Cobalt oxides and hydroxides; commercial cobalt oxides	20121930	2822 00 00	Eurostat, UNC
Processed	Cobalt chlorides	20133134	2827 34 00	Eurostat, UNC
			2827 39 30	
Processed	Sulphates of cobalt and of titanium	20134162	2833 29 30	Eurostat
Processed	Cobalt acetates	–	2915 23 00	Eurostat, UNC
Processed	Refined cobalt	–	–	BGS, USGS

### 2.2 Long-term statistical data

Long-term statistical data was explored and afterwards acquired according to its availability, for the domestic production and international trade of the different commodities containing Co. In this section, the steps of data exploration, acquisition, processing, and analysis are described.

#### 2.2.1 Exploration

As Fig. 1 presents, a number of open data sources were consulted for the data exploration. The domestic production of commodities was explored in the British Geological Survey (BGS), the US Geological Survey (USGS), and the Austrian Federal Ministry of Agriculture, Regions and Tourism (BMLRT).

From the BGS, the World Mineral Statistics (BGS 2020) was consulted (including the archives from 1910s to 1970s), which provided data about the mine production of Co (primary) and refined (processed) Co per country in tonnes of metal, from 1941 to 1938, respectively, until 2018. From the USGS, the National Minerals Information Center was consulted (USGS 2020). This source provided mostly information and data related to the domestic production and international trade of the USA, but also about the main global producers of primary and processed Co. The information was available in tonnes of metal from 1962 to 1948, respectively, until 2016. The World Mining Data report of the BMLRT (BMLRT 2020) provided values for the production of primary Co in tonnes of the metal per country, from 2007 to 2016.

For the international trade of commodities, data was explored per country consulting the UN Comtrade database (UNC) (UN Comtrade 2020), and the Eurostat database (Eurostat 2020). From both sources, data was available in mass and/or monetary value (US dollar and euro, respectively) from 1988 to 2018. The Finnish customs database (ULJAS) (ULJAS 2020) was also consulted for the Finland's trade of the commodity “Cobalt mattes and other intermediate products of cobalt metallurgy; unwrought cobalt; cobalt powders”, which also reported data in mass (from 2005 to 2018) and euros (from 1995 to 2018). The BGS and USGS provide information about international trade but only in global terms; therefore, these sources were not considered since it is not possible to differentiate between trade intra-EU and extra-EU.

#### 2.2.2 Acquisition

Data was acquired after comparing the available data from the different explored sources, from the earliest to the latest available years. The data was obtained for each one of the 27 member states and each commodity.

For the domestic production of primary and processed Co, data in mass value was acquired from the BGS and the USGS. The data from the BMLRT for primary Co was highly similar to the data from the BGS, but for a shorter time window. For these reasons, it was not included in the analysis.

Regarding the international trade of commodities, data in mass and monetary value was acquired from Eurostat and UNC databases. They showed strong differences between the reported values. In addition to these differences, it was observed that UNC did not report values for the commodities “Ash and residues containing mainly cobalt” and “Sulphates of cobalt and of titanium”. Furthermore, Eurostat and UNC also presented hidden or aggregated categories, named “confidential trade” and “special category” respectively, mostly related to the trade of the commodity “Cobalt mattes and other intermediate products of cobalt metallurgy; unwrought cobalt; cobalt powders”. Because of this, the ULJAS database was also consulted, in order to complete the flow of the international trade of the mentioned commodity. It is important to point out that the UNC database also presents internal differences of the data, depending on if the member state is considered a reporter or a partner. Because of this, data was acquired double from UNC, considering the member states as reporters and as partners (henceforth named UNC as reporter and UNC as partner, respectively). The acquired values are presented in the SI.

#### 2.2.3 Processing

Some of the acquired data had to be processed in order to obtain long-term historical values of the domestic production and international trade in metal content, as the trade of commodities was reported in total mass (not in Co content) and/or monetary values. Due to the identification of hidden flows, different ratios were estimated to first obtain all values in mass, which were then transformed into Co mass values. These hidden flows were related to two types of data gaps: data only reported in monetary value, and data reported as “special category”. The domestic production of primary and processed Co was already reported in mass of the metal; therefore, no data processing was required.

• Data in monetary value: From the databases, most of the data was reported in mass and monetary value, but in some cases only the monetary value was given. In that case, the data was converted to mass value through a ratio mass/currency, which was calculated per commodity using data for the same member state and the same year, based on the total trade of the commodity (intra and extra EU). When there was insufficient data to use this method, the ratio mass/currency was calculated for the trade of the EU for the same year, based on the information of the other member states. This ratio was calculated using the data of each source, i.e., the data of the different sources was not mixed. In the case of UNC, the ratio was calculated separately using the data of the member states as reporters and as partners. To transform the data from ULJAS, a mass/currency ratio was calculated examining the data from 2005 to 2014 (last 10 years where data in mass is available), which was reported in mass and euros. Then, these values were graphically compared to the Co market price per year. Based on this comparison, a mass/currency ratio was established per year from 2015 to 2018 according to the trend of the graph, in order to transform monetary values into mass equivalent. Initially, the approach of Matos et al. (2020) was followed, who made use of an average value for the mass/currency ratio for the involved years. This approach seems a reasonable proxy for the period 2015–2017; however, it might overlook the substantial increase of the Co price in 2018.
• Data as “special category”: The second type of hidden flow was related to the data reported as “special category” by UNC. This flow was recorded as global trade (i.e., total import from or export to all countries, categorised as “world”); therefore, it was necessary to find a way to estimate how much was traded intra and extra EU. To do so, the ratio between the trade extra EU and the global trade was estimated, considering the data reported in monetary value since it was more complete. The ratio was calculated based on the data of each member state, calculating an average value per year.

Next to these two types of hidden flows, we also have to consider the hidden flow coming from the incomplete data of Eurostat and UNC for the commodity “Cobalt mattes and other intermediate products of cobalt metallurgy; unwrought cobalt; cobalt powders”, which was complemented with data from the ULJAS database. Thus, in total three types of hidden flows were considered.

Finally, the data reported in and transformed to mass was converted to mass of Co multiplying by the Co content of the different commodities. Estimated or average values were used, which are presented in Table 2. To estimate the Co content of the commodity “Cobalt mattes and other intermediate products of cobalt metallurgy; unwrought cobalt; cobalt powders”, a ratio was calculated between the trade in monetary value and the value in mass. This value was compared to the Co price reported per year, which corresponds to the price of high purity Co (Metalary, 2020). It was assumed a Co content of 100% for ratios equal or above the price, which was considered processed Co (named “Co powders”). For ratios below the price, it was assumed a content of 17% of Co following European Commission (2020c); considered as semi-processed Co (named “Co mattes and intermediates”). If the trade of this commodity was only reported in monetary value for a member state, the ratio of traded semi-processed Co and processed Co was calculated from the results for the whole EU, to transform the data to mass value.

**Table 2:** Estimated or average Co content of the different commodities.

Co content	Value (wt%)	Comment	Reference
Co ores and concentrates	10	Typical value	Darton Commodities Ltd. (2018)
Co waste and scrap	19	Average value	Ferron (2013), Allstar Magnetics (2018), Adams (2019)
Ash and residues of Co	0.4	Average value	Vítková et al. (2013)
Waste and scrap of Ni alloys	4	Average value	Roskill (2014)
Co mattes and intermediates	17	Assumption based on the Co content of crude oxides and hydroxides	European Commission (2020c)
Ni mattes	0.5 - 5	Depending on the country of origin	Roskill (2014)
Co oxides and hydroxides	70	Calculation based on elementary composition	–
Co chlorides	25	Calculation based on elementary composition	–
Sulphates of Co	20	Calculation based on elementary composition	–
Co acetates	21	Calculation based on elementary composition	–
Co powder	100		European Commission (2020c)

#### 2.2.4 Analysis and results

To estimate the domestic production and international trade at the EU level, the values estimated for the different member states were added up per year. To do so, only the production of member states and the trade of member states with non-member states were considered (extra EU trade). As it was already indicated in Section 2.1, the member states were considered from before they joined the EU.

To take into account the uncertainty of the calculations, two sensitivity analyses were carried out. The first one is related to the commodity “Sulphates of Co”, since Co traded as sulphates was estimated from the commodity “Sulphates of cobalt and titanium”. To estimate how much it corresponds to Co, it was assumed that 50% were sulphates of Co and 50% sulphates of titanium. This assumption was taken from European Commission (2020c). In order to assess how this assumption affected the results, a sensitivity analysis was performed using a relation 25/75 and 75/25 between the sulphates of Co and the sulphates of titanium. The second sensitivity analysis was carried out for the data reported in monetary value. As it was explained before, when data was only reported in monetary value a ratio mass/currency was calculated based on the average price of the same commodity for the same member state or for the EU, depending on the available data. For the sensitivity analysis, the minimum and maximum presented by the member states for each year was considered for the ratio. The results from both sensitivity analyses are presented in the SI and discussed in Section 3.

Final datasets for the domestic production and international trade of the Co-containing commodities were obtained from the analysis of the acquired and processed data, covering different timeframes depending on the source, which are presented in the following section.

## 3 Results and discussion

In this section, the final reconstruction of the domestic production of primary Co and processed Co, and the international trade of a number of Co-containing commodities is presented. In addition, the contribution of hidden flows to the trade is analysed. The results correspond to processed data; the raw data is presented in SI. Table 3 gives an overview of the final dataset according to the type of flow (domestic production or international trade), commodity category, timeframe, and source.

**Table 3:** Overview of final datasets. BGS: British Geological Survey, USGS: US Geological Survey, UNC: UN Comtrade, ULJAS: Finnish customs database.

Flow	Commodity category	Available timeframe (Source)
Domestic production	Primary	1962–2016 (USGS) / 1941–2018 (BGS)
Domestic production	Processed	1948–2016 (USGS) / 1938–2018 (BGS)
International trade	Primary	1988–2018 (Eurostat and UNC)
International trade	Semi-processed	1988–2018 (Eurostat and UNC) / 2005–2018 (ULJAS)
International trade	Processed	1988–2018 (Eurostat and UNC)
International trade	Secondary	1988–2018 (Eurostat and UNC)

### 3.1 EU domestic production

**Fig. 3:**
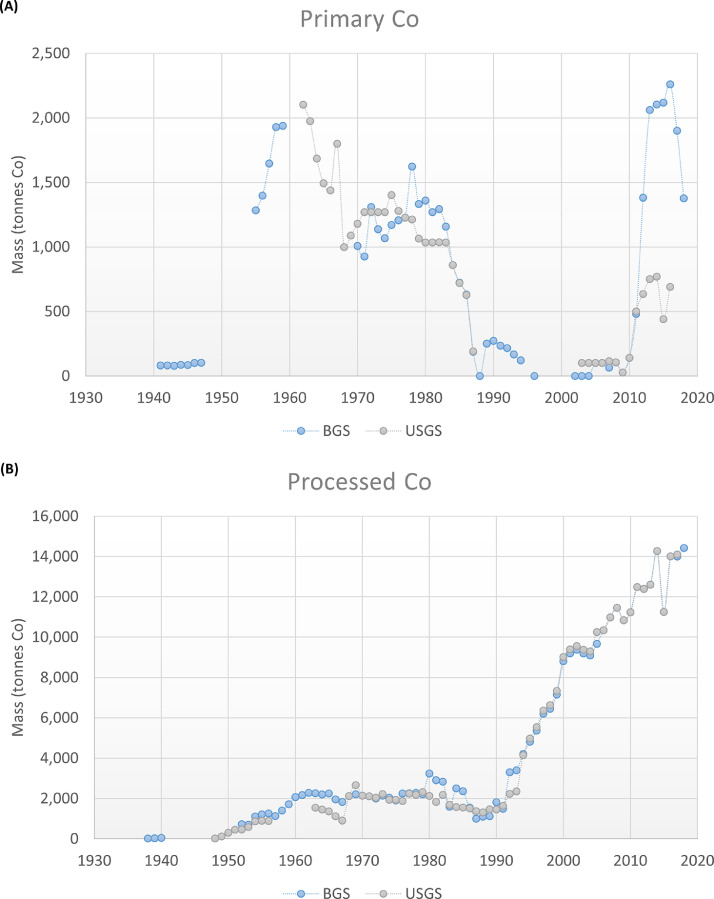
Domestic production of primary and processed Co over time for the EU-27.

The domestic production of primary and processed Co of the EU-27 is presented in Fig. 3. The data was obtained from the BGS and the USGS databases. BGS reports for the periods 1941–2018 and 1938–2018, for primary and processed Co, respectively; and USGS for the periods 1962–2016 and 1948–2016, respectively. The discontinuity of the graphs is due to data unavailability.

For primary Co, the data corresponds to the domestic production of Finland. For the period 1941–1944 Germany and Italy also reported some minor production, but from the source it is not clear if it corresponds to primary Co; therefore it was not considered in the analysis. In addition, it is reported that Poland and Bulgaria also produced ores containing Co, but that it was not recovered, or that the information is inadequate for reliable estimates (Shedd, 2019; BGS, 2020). From Fig. 3(A), it is observed that before 1955 the reported production of primary Co is negligible. When comparing the two sources, the reported data deviate from each other. In general (when data is available), the BGS reports higher values than the USGS. The strongest differences are for the period 2011–2016, where the BGS reports values about three times the values reported by the USGS. A plausible reason of these differences is that the reported values correspond to recovered Co in some cases, and to recoverable Co in others. For instance, the BGS indicates that the values from 1992 to 2012 correspond when possible to recovered Co, and that frequently the values present a considerable disparity between the Co content of ore raised and Co actually recovered. After 2012, the values relate either to recovered Co or to Co contained in ore raised. Meanwhile, from 1962 to 1984 the values reported by the USGS represent recovered Co; afterwards, they represent recoverable Co. This difference is related to the fact that Co is mainly obtained as a by-product of nickel and copper, while only a small share is from direct primary Co production (Petavratzi et al., 2019). Because of this, the Co market has been and still is highly dependent on the copper and nickel market (Mudd et al., 2013; van den Brink et al., 2020). This explains why even if cobalt-containing ores are mined, Co is not necessarily recovered. It is also one of the reasons of data gaps about Co production, since it is not systematically clear how much Co is actually recovered from the mining operations.

It is also observed that in the period 1988–2010, almost no Co was mined. According to the USGS, in 1983 the Luikonlahti mine (Finland) ceased its mining operations (Kirk, 1985), and in 1988 the state-owned Co and nickel producer, Outokumpu Oy, started the closing of its copper-cobalt mine, due to the Co production had become unprofitable (Kirk, 1989). In 1989, the mine was closed, after being opened for 76 years. It was estimated that during this period the mine produced 60,000 tonnes of Co in concentrates (Shedd, 1990; 1991).

Only in the middle of the 2000s, new mining projects started to emerge, which explains the increasing production of primary Co after 2010. In 2005 and 2006, the feasibility study of the Kylylahti copper-cobalt-nickel-gold deposit and two polymetallic sulfide deposits were carried out. These studies resulted in the Kylylahti and Sotkamo mines, which started to operate in 2012 and 2008, respectively. In 2007, the company Belvedere Resources Ltd. started to mine from its Hitura and Sarkiniemi nickel-copper sulfide mines, but by the end of the year they were placed on care-and-maintenance status in response to low nickel prices. In 2009, the Hitura mine was restarted, ceasing its production in 2013. In 2012, First Quantum Minerals Ltd. completed the construction of its Kevitsa open pit nickel-copper-platinum group metals (PGM) sulfide mine and beneficiation plant and began commercial production (Shedd, 2007, 2008, 2010, 2012, 2015, 2016).

**Fig. 4:**
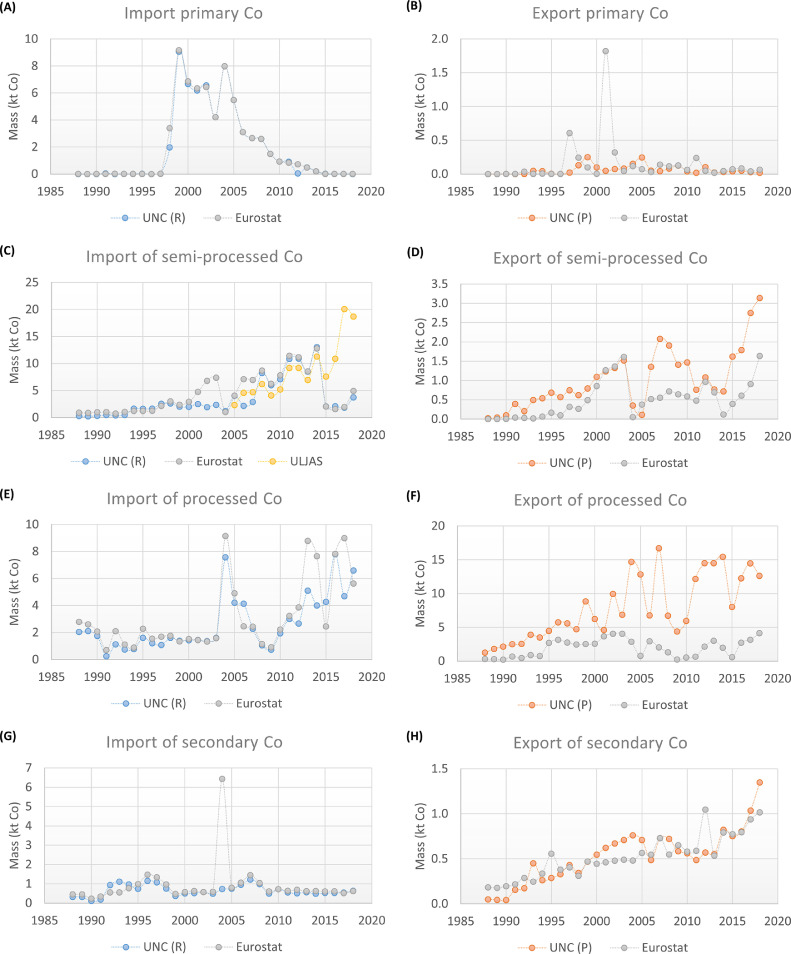
Import in and export from the EU-27 of primary, semi-processed, processed, and secondary Co according to the UN Comtrade (UNC) and Eurostat databases, between 1988 and 2018. UNC (R): member states as reporters, UNC (P): member states as partners. ULJAS database was also consulted for the import of Co mattes and intermediates to Finland.

In comparison to the reported values of primary Co, the values of processed Co (Fig. 3(B)) present very little differences between the sources. The strongest differences are for the period 1980–1992, where in general the BGS presents higher values. From the figure, it is observed that between 1970 and 1990, the production of processed Co was relatively constant, around 2000 tonnes of Co per year. In the middle of the ’90s, the processed Co demand started to increase due to an increasing demand of rechargeable batteries (Shedd, 1997). To cover this increasing demand, the EU had to rely on imports of Co. As it is observed in Fig. 4, the periods 1990–1997 and 2009–2018 were mostly covered by a net import of semi-processed Co. Between 1998 and 2008, there was in addition a net import of primary Co.

When analysing the global production of refined Co, it is observed that the EU domestic production follows a similar trend (BGS, 2020).

According to the BGS and the USGS, the refining of Co has taken place in Belgium, Finland, France, and Germany. For Germany (Federal Republic of Germany between 1949 and 1990), there are reported values between 1948 and 1991. Finland, France and Belgium report values starting in 1967, 1968, and 1992, respectively, continuing until today. However, according to the USGS (Ware, 1964; DeHuff, 1969) during the 60s’, Belgium and Italy also refined Co (salts and oxides, and metal, respectively).

Between 1948 and 1961, Germany refined concentrated Co coming mostly from Finland (refinery of Duisburger Kupferhütte), and treated Co-bearing scraps and residues at the refinery of Gebrüder Borchers A.G (Davis and Buck, 1953; Bilbrey, 1961). In 1962, the latter also started to treat spent catalysts (Bilbrey and Clarke, 1962). In 1968, Outokumpu Oy started to operate its Kokkola refinery in Finland, processing the concentrates that used to be shipped to Germany (DeHuff, 1969).

From that year onwards, Finland has been one of the main refiners of Co of the EU. The Kokkola refinery (nowadays owned by Umicore) has been the main refinery of the country, producing metal powders, briquettes, oxides, and compounds. Another refinery, called Harjavalta, refined Co since 1996, producing Co sulphates in the last years (Shedd, 2019).

Regarding France, the reports mainly mention the Saundoville electrolytic refinery (nowadays owned by the Eramet Group), where Co chlorides are produced (USGS, 2020).

For Belgium, the oldest found report of refinery is from 1968, where the Metallurgie Hoboken S.A is mentioned as a producer of Co salts and oxides. In 1986, the company started to treat Co-containing scrap and spent catalysts (DeHuff, 1969; Kirk, 1988). In 1992, Union Minière merged with its subsidiaries Metallurgie Hoboken-Overpelt, Vieille-Montagne and Mechim, changing its name to Umicore in 2001 (Umicore, 2020). In the same year, the company compensated supply problems by diversifying its supply sources, recovering Co from secondary materials at full capacity (Shedd, 1994). Its operation and treatment of secondary material continues until today producing metal powders, oxides, salts, and compounds (Shedd, 2012). It is important to indicate that between 1992 and 1999, the Belgian reported values correspond to the annual refinery capacity, which is the maximum quantity of product that can be produced on a normally sustainable long-term operating rate; however, actual production might have been lower. In addition, for Fig. 3 the annual capacity was considered instead of the reported values from 2003 onwards (for both, BGS and USGS), since the latter included the production from outside the EU, e.g. Umicore's plants in China and South Africa (Shedd, 2005; Darton Commodities Ltd., 2018).

### 3.2 EU international trade

The international trade of Co-containing commodities for the EU-27 is shown in Fig. 4. The imports and exports were aggregated per category of commodity (primary, semi-processed, processed, and secondary material). The figure presents the processed data for the period 1988–2018. The full detail for each commodity is presented in SI.

Before discussing the results for each commodity category, a general remark has to be given. From all figures, it is observed that the data from UNC changes substantially depending on if the member states are considered reporters or partners of the trade. This difference is because in UNC, imports are recorded as CIF (cost insurance and freight) while exports are FOB (free on board). According to the World Bank, this may represent a 10 to 20% difference, with import values normally higher than export values. For a given country, imports are usually recorded with more accuracy than exports because imports generally generate tariff revenues while exports do not (UN Trade Statistics, 2009; World Bank, 2010). Therefore, the value obtained of the imports with members states as reporters, and of the exports with member states as partners, are expected to be more accurate. Hence, the imports were considered with the member states as reporters, and the exports with the member states as partners for the reconstructed datasets from UNC. The following discussion was developed accordingly (the full dataset and related discussion is presented in SI). This does not only allow having a single dataset, but also removing some outliers from it.

For a more detailed discussion, the rest of this section is organised per category of commodity.

#### 3.2.1 Primary Co

This category considers the commodity “Co ores and concentrates”. For the imports (Fig. 4(A)), the data from Eurostat and UNC is mostly the same. For the exports (Fig. 4(B)), the data from both datasets follow a similar trend, although they differ strongly in some points.

When analysing the data of the import from both datasets, it is observed that the trade increases after 1997, with an average import of 5.3 ktonnes of Co between 1998 and 2008. This corresponds with an expansion of the Kokkola and Harjavalta refineries in Finland, which finished between 1995 and 1996 (Shedd, 1997). After 2008, the imports present a steady decrease, until the present year, where almost no primary Co is being imported to the EU. The import of this material has been replaced mostly by the import of semi-processed material (see Fig. 4(C)).

The export in turn, when analysed with the EU as partner, has fluctuated between 0 and 250 tonnes of Co per year. For the period 1997–2008, it represented 2% in average of the imports. In 2018, the export of primary material was less than 70 tonnes of Co.

Two outliers are observed for the export of primary Co, both recorded by Eurostat. The first one is for 1997, where an export of 4 ktonnes (0.4 ktonnes of Co according to the estimations) is reported from Greece to Morocco. The second outlier occurs in 2001, where an export of 18 ktonnes of material (1.8 ktonnes of Co according to the estimations) is reported from Greece to Russia. It is possible that these exports come from the nickel production that takes place in Greece. According to Melfos and Voudouris (2012), Greece was the major nickel supplier in EU in those years, recovering the metal through pyrometallurgical methods. The ores also contained cobalt, which could be separated by hydrometallurgy; however, the metal was not recovered in the country (Melfos and Voudouris, 2012). It is curious, however, that this flow would be reported as “Co ore and concentrate” and not as “nickel ores and concentrates” (HS 260,400) or “ferronickel” (HS 72,026,000). A plausible explanation would be that the Co concentration was very high in those years, and might have been exported to Russian and Morocco's refineries under the code of “Co ore and concentrate”. This is a clear example of how much of Co trades are unknown or missing due to its by-product nature and the lack of more detailed data and information.

Analysing each member state, the main importer of primary Co has been Finland according to both sources. For the export, the main member states have been Belgium and the Netherlands according to UNC, and Greece and Belgium according to Eurostat (for more details see SI). It is worthy to note that for primary Co (and also other of the Co-containing commodities) the Netherlands is presented as one of the main importers or exporters. However, as it was seen in the previous section, this country is not involved in the primary production or refinement of Co. Probably, its role in the metal trade is due to the fact that the main port of the EU is located in the city of Rotterdam.

#### 3.2.2 Semi-processed Co

In this category two commodities are considered, “Co mattes and intermediates”, and “Ni mattes”. For the whole study period (1988–2018), the imports (Fig. 4(C)) and exports (Fig. 4(D)) present a general increasing trend. For the imports, the data from Eurostat and UNC is highly similar, although between 2001 and 2007 data from Eurostat is considerably higher. For the exports, the data from both datasets follow a similar trend, even though for many points the values differ strongly between one and another dataset.

As it was mentioned in Section 2.2, it was found that Eurostat categorizes some of the trade as confidential. This is the case for the commodities that are part of the groups with heading code 75 and 8105. Heading code 75 is related to “Ni mattes”, with the group code 75SSS284, which is described as “Confidential trade of chapter 75 and Standard International Trade Classification (SITC) group 284 (1997–2500)” (SITC group 284 corresponds to Nickel ores & concentrates; nickel mattes, etc.). Heading code 8105 is related to “Cobalt mattes and other intermediate products of cobalt metallurgy; unwrought cobalt; cobalt powders”, with the group 8105S689 “Confidential trade of heading 8105 and SITC group 689 (1999–2500)” (SITC group 689 corresponds to Miscellaneous non-ferrous base metals for metallurgy). As the information is not publicly available, it is not possible to know to which extent it affects the calculated trade. However, in order to include these hidden flows and estimate the trade as complete as possible, data from ULJAS was used to complement the data from Eurostat and UNC, specifically the import of the commodity “Co mattes and intermediates”, even though it only considers the imports of Finland. Between 2005 and 2014, the data reported by ULJAS agrees with the data of Finland from Eurostat and UNC as reporter. However, from 2015 onwards, the data is not available in the Eurostat and UNC databases. The data from ULJAS shows that the imports of semi-processed material increases substantially between 2015 and 2018, agreeing with the decrease of the import of primary material.

Regarding the exports, a steep decrease is observed between 2003 and 2005. This is explained by different reasons. Eurostat on one side does not report any values for Finland's export of “Co mattes and intermediates” between 2004 and 2009. UNC in turn reports data, but according to the applied data processing, most of the export corresponded to Co powders; hence, categorised as processed Co. In addition, it was not possible to complete the export of this category with data from ULJAS, since the available information of the database is shown in monetary value.

When analysing the single commodities considered as semi-processed Co (“Co mattes and intermediates”, and “Ni mattes”), it is observed that between 1988 and 2000 both commodities were imported at a similar extent, but from 2000 onwards, “Co mattes and intermediates” became the main imported commodity of this category. For the export, between 1988 and 2005 the main exported commodity was “Co mattes and intermediates”, but after 2005 the export of “Ni mattes” shows an increasing trend, being in 2017 and 2018 the main exported commodity of this category.

According to the data from UNC, the main importers of semi-processed Co have been Finland, France, and the Netherlands; and the main exporters Belgium, Finland, Germany, and the Netherlands. Eurostat in turn reports Finland, France, and the Netherlands as main importers; and Belgium, Czech Republic, France, Germany, the Netherlands, and Sweden as main exporters.

#### 3.2.3 Processed Co

The processed Co includes four or five commodities depending on the source: “Co powders”, “Co oxides and hydroxides”, “Co chlorides”, “Co acetates”, and “Sulphates of Co” (the latter only reported by Eurostat).

For the import (Fig. 4(E)), it is observed that the data from both sources have a similar trend, although in general the data from Eurostat is higher. For the export (Fig. 4(F)), a clear difference is observed among the data sources; UNC presents higher values for almost every year of the studied period. The highest difference is for the year 2007; according to UNC, Belgium exported around 14 ktonnes of Co as oxides and hydroxides, while Eurostat does not report any export of this commodity for that year. According to UNC, most of the export was to Malaysia and the Republic of Korea. Unfortunately, just from the data it is not possible to establish the reasons of this difference. It is important to remember that from the Eurostat database the data is reported by the EU-member states, but that from the UNC database the data is reported by countries that imported from the EU-member states (extra-EU countries), which could apply different methodologies to record the data. Nevertheless, a specific reason cannot be found in the current scope of this work.

Regarding the single commodities, “Co oxides and hydroxides” and “Co powders” are the two main traded commodities of this category. The other three commodities (Co chlorides, Co acetates, and Sulphates of Co) are traded in a much lower extent. In average for the studied period, the import and export of these three commodities represents 6 or 12% and 2 or 15% of the total trade according to UNC and Eurostat, respectively. However, it should be noted that according to Eurostat, for the year 2010, these commodities accounted for 37% of the total import.

A number of member states have been involved in the trade of these four or five commodities along the studied period. According to the data of UNC, Belgium and Finland have been the main exporters; for the import, several countries have played a role, such as Belgium, France, and the Netherlands. Eurostat reports that some of the main exporters have been Belgium, Finland, and the Netherlands; and that Belgium, France, and the Netherlands have been some of the main importers (for more details see SI).

#### 3.2.4 Secondary Co

This category comprises the commodities “Co waste and scrap”, “Waste and scrap of Ni alloys”, and “Ash and residues of Co” (the latter only reported by Eurostat). The latter is not considered in the analysis, since its trade is very low compared to the other two commodities (less than 2% in average along the studied period).

In general terms, a relatively constant import (Fig. 4(G)) (around 0.7 ktonnes of Co) and an increasing export (Fig. 4(H)) is observed for this category.

For the imports, an outlier is observed for the year 2004, recorded by Eurostat. It comes from the import of “Co waste and scrap” to France from the UK. The imported amount was 29.3 ktonnes of material; considering 19% of Co content, the estimated amount of imported Co corresponds to 5.6 ktonnes. In comparison, the UNC does not report any import of Co waste and scrap to France from the UK in 2004 with France as reporter, while as partner, it reports a similar monetary value to the one reported by Eurostat but a mass value 99% lower (import 0.1 ktonnes of material as partner according to UNC).

Leaving the outlier aside, most of the imported and exported Co as secondary material comes from “Ni waste”. For the studied period, an average of 0.6 and 0.4 ktonnes of Co have been imported and exported, respectively, in Ni waste.

According to the different sources, several countries have played a role in the trade of secondary Co, among them France, Germany and the Netherlands (for more details see SI).

### 3.3 Contribution of hidden flows to the trade

**Fig. 5:**
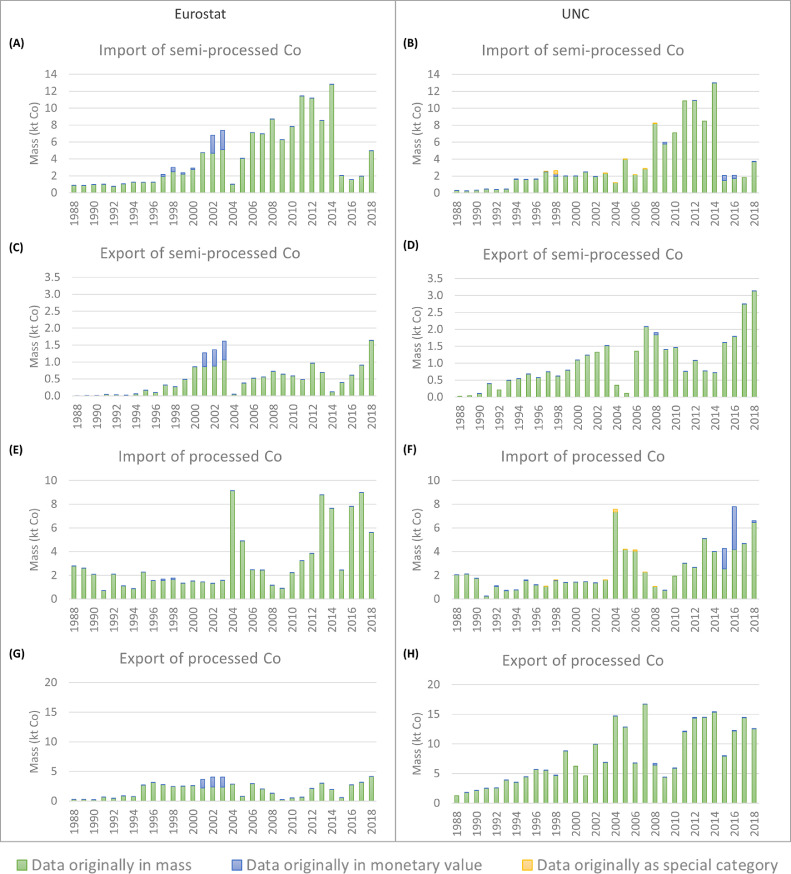
Contribution to the results of the trade of semi-processed and processed Co of the data originally reported in mass, in monetary value, and as "special category" for Eurostat and UN Comtrade (UNC).

The contribution of the estimated hidden flows to the international trade of Co-containing commodities is presented in Fig. 5. Since the contribution of the hidden flows coming from the incomplete data of Eurostat and UNC for the commodity “Cobalt mattes and other intermediate products of cobalt metallurgy; unwrought cobalt; cobalt powders” was already discussed in Section 3.2, here only the hidden flows related to data reported in monetary value only and data reported as “special category” are analysed.

For the commodities categorised as primary and secondary Co, the data was reported mostly in mass, hence, the contribution of the data originally reported in monetary value or as “special category” to the final results was marginal (see SI). This means that no significant hidden flows were found for these commodities, the reason why primary and secondary Co are not included in Fig. 5.

For semi-processed Co (Fig. 5A–D), hidden flows were found regarding the two types of data gaps, although the contribution to the results of the data originally reported as “special category” is small. As Fig. 5(A) and (C) present, the highest contribution was for the Eurostat dataset, where most of the relevant data related to the commodity “Co mattes and intermediates” was originally reported in monetary values between 1997 and 2003. For these years, the data in monetary value accounts for 8 to 37% of the estimated trade. For instance, for the year 2003 the estimated imported mass of the commodity was 13 ktonnes of material, from which 2.3 ktonnes correspond to Co according to the calculations.

Finally, hidden flows were identified for the category of processed Co (Fig. 5E–H), mostly related to data originally reported in monetary value. Once again, the contribution of the data originally reported as “special category” is negligible. From Eurostat, for the years 2001 to 2003, around 40% of the exported “Co oxides and hydroxides” was estimated from data reported in monetary value. From UNC, it mainly affected the final import of “Co powders” for the years 2015 and 2016. In 2015, it was estimated that 3.5 ktonnes of Co were imported as “Co powders”, of which 1.7 ktonnes (49%) were estimated from data reported in monetary value. For the year 2016, the hidden flow accounted for 52% of the imported Co in “Co powders”.

Nevertheless, it is important to keep in mind that the values above mentioned were obtained through the estimated mass/currency ratios, which vary in a broad value range (see SI). When looking at the sensitivity analysis (see SI), it is observed that the results could change drastically. For example, for semi-processed Co, the contribution of hidden flows to the imports according to the UNC dataset could be from 12 to 2600 times higher than in the base case for the years 2015–2018, when using the maximum value of the ratio. For the export of semi-processed Co and processed Co, for the period 2001–2003 the contribution of hidden flows could be between 4 and 22 times, and between 4 and 49 times higher, respectively, compared to the base case according to the Eurostat database, when using the maximum value. This is a clear example of how the lack of more transparent and detail data contributes to the uncertainty of the results.

### 3.4 Limitations and future research

Despite our efforts to make the presented study as complete as possible, limitations were encountered in this work. First, there are different sources of uncertainty. For instance, the Co content of the different commodities are based on estimations or average values. Another example are the ratios (e.g., “trade extra EU/global trade” ratio, “mass/currency” ratio) that were applied to obtain the final results. The value of these ratios was estimated based on the available data, but some assumptions were made since a range of plausible values could be used. We tried to understand the impact of some of these uncertainty sources through the sensitivity analysis, keeping all the limitations in mind.

Second, data gaps were found in some of the datasets, which we tried to complement. Nevertheless, it was not possible to fully establish the extent of hidden flows that are reported as “confidential trade” and their impact on the results. In particular more transparent data about the commodity “Cobalt mattes and other intermediate products of cobalt metallurgy; unwrought cobalt; cobalt powders” is required, giving its crucial role in the Co value chain in the EU, in order to depict and understand in a more accurate the societal metabolism of Co.

Third, through the present analysis it was not always possible to establish probable reasons for the differences between the data reported by one or another data source. Furthermore, the scope of the study was to analyze the trade of Co from the perspective of the EU, but the partners were not analysed in detail. Finally, the scope of this work was focused on Co flows, but Co stocks were not addressed.

Some of these limitations may be tackled in future studies. One example is the analysis of the trading routes of the different Co-containing commodities intra-EU and extra-EU, which could be relevant for policy and for responsible sourcing. This could also help to elucidate why there are differences between the datasets. Another future study is the analysis of the dynamics of historical stocks and flows of Co in the EU.

Regarding the data gaps and data uncertainty, substantial improvements are needed, mainly from governmental statistical bodies and industries to report more transparent and detailed data (e.g., not only economic values but also specific in terms of physical quantities and composition), which would certainly be beneficial for all stakeholders.

## 4 Conclusions

Long-term statistical data was explored and analysed to assess the historical domestic production and international trade of a number of cobalt-containing commodities for the EU, assessing each member state including before they joined the EU. Several open data sources (e.g., the British Geological Survey (BGS), the US Geological Survey (USGS), the Eurostat database, and the UN Comtrade (UNC) database) were explored for data from 1900 onwards. The earliest data was found for 1938, and the latest for 2018.

The presented analysis brings different novelties. First, to the best of the authors’ knowledge, it corresponds to the first long-term analysis up to the present day of the trend of Co flows in the EU. Second, the analysis was based on a number of official data sources such as geological surveys, identifying strong differences between the reported data. Large differences exist between the data reported by the member states as reporters and as partner of the trade. It is clear that even from the most reliable data sources such as Eurostat and UN Comtrade, strong differences in the data can be found; therefore, researches have to be careful when using this type of data in their studies. Preferably, data from different sources should be consulted. Finally, despite the difficulties encountered when interpreting the dynamics of the production and trade flows, which are often discontinue or un-transparently reported, hidden flows were also identified and analysed. Through the conversion of data originally reported in monetary values and as “special category”, and the exploration of the Finnish customs database (ULJAS), the Co flows were depicted in a more complete way. With the applied methodology, it was possible to complete up to more than 50% of the flows of specific years.

The presented analysis could be used to further study the long-term historical societal metabolism of Co in the EU, through tools such as material flow analysis (MFA), similarly to what Sun et al. (2019) developed at a global scale, or Soulier et al. (2018) developed for copper at the EU level. The results exposed in this manuscript could enable better estimations of the dynamics of historical stocks and flows of Co in the EU, which to date, has been done based on rough estimations of past trends, and where the difference between datasets have not been taken into account. Such study will be subject of further research, where a retrospective dynamic MFA can be envisaged in order to estimate the historical stocks and flows of Co in the EU over a long-term period. A clear understanding of the importance of historical flows is crucial to quantify historical and actual anthropogenic stocks properly, which in turn are key for a correct management of the metal. For instance, potential sources of secondary Co can be identified more accurately, promoting the circularity of the metal in the techno sphere. In addition, a proper understanding of current stocks is needed in prospective assessments, which can be of value in policy support.

## CRediT authorship contribution statement

**María Fernanda Godoy León:** Conceptualization, Formal analysis, Investigation, Methodology, Visualization, Writing – original draft, Writing – review & editing. **Gian Andrea Blengini:** Writing – review & editing. **Jo Dewulf:** Conceptualization, Supervision, Writing – review & editing.

## Declaration of Competing Interest

The authors declare that they have no known competing financial interests or personal relationships that could have appeared to influence the work reported in this paper.

## Appendix Supplementary materials

Image, application 1

Image, application 2
