# Supplementary material for: Analysis of long-term statistical data of cobalt flows in the EU
Source: Resour Conserv Recycl. 2021 Oct;173:105690. doi: 10.1016/j.resconrec.2021.105690 (PMC8291040; doi:10.1016/j.resconrec.2021.105690)
Supplement: Supplementary file 1 [file mmc1.docx]

Analysis of Long-term Statistical Data of Cobalt Flows in the EU

María Fernanda Godoy ^a^, Gian Andrea Blengini ^b^, Jo Dewulf ^a^

^a^ Ghent University (Belgium), ^b^ Joint Research Centre (Italy)

SUPPORTING INFORMATION A

Content

[1. Raw data for the EU 2](#_Toc65850972)

[2. Processed data for the EU 2](#_Toc65850973)

[3. Results per commodity 4](#_Toc65850974)

[4. Results according to main member states 7](#_Toc65850975)

[6. Sensitivity analysis for data originally in monetary value 13](#_Toc65850976)

[7. Sensitivity analysis for sulphate of cobalt 16](#_Toc65850978)

# Raw data for the EU

See SI-B.

# Processed data for the EU

*Primary Co*

For both, imports and exports, the data from Eurostat and UNC as reporters is mostly the same. The data from UNC as partners, however, is highly different, being in general, lower than the other two datasets. For the import, the internal difference of the data from UNC is clearly more than 10-20%.

*Semi-processed Co*

For the imports, an outlier is shown for 2009, which corresponds to imports of Ni mattes to France from New Caledonia, with France as partner. The imported amount was 682 ktonnes of mattes, which corresponds to 34 ktonnes of Co considering a Co content of 5%. The same import, considering France as reporter, was around 70% less.

*Processed Co*

For the import, it is observed that the data from the three sources have a similar trend. For the year 2004, it is observed that the value from UNC(P) presents a peak around 65% higher than the value from UNC(R), and 36% higher than the value from Eurostat. This is mainly due to differences on the trade of Co powders reported for/by Belgium. It is important to remember that the trade of Co powders was estimated from the trade of the commodity “Cobalt mattes and other intermediate products of cobalt metallurgy; unwrought cobalt; cobalt powders”. For Belgium, the UNC as reporter and Eurostat report zero and about 1 ktonne of imported material respectively, while UNC as partner reports around 5.4 ktonnes, of which 4.6 ktonnes were estimated as Co.

Regarding other commodities of this category, it is observed that Co oxides and hydroxides do not present strong differences between the data reported by the different sources, except for the year 2018, where the data from Eurostat and UNC as reporter is about four times the data reported by UNC as partner.

*Secondary Co*

For both, imports and exports, the data from Eurostat and UNC (as reporters and as partners) is highly similar. For the imports, an outlier is observed for the year 2004, recorded by Eurostat, which is explained in the main text.


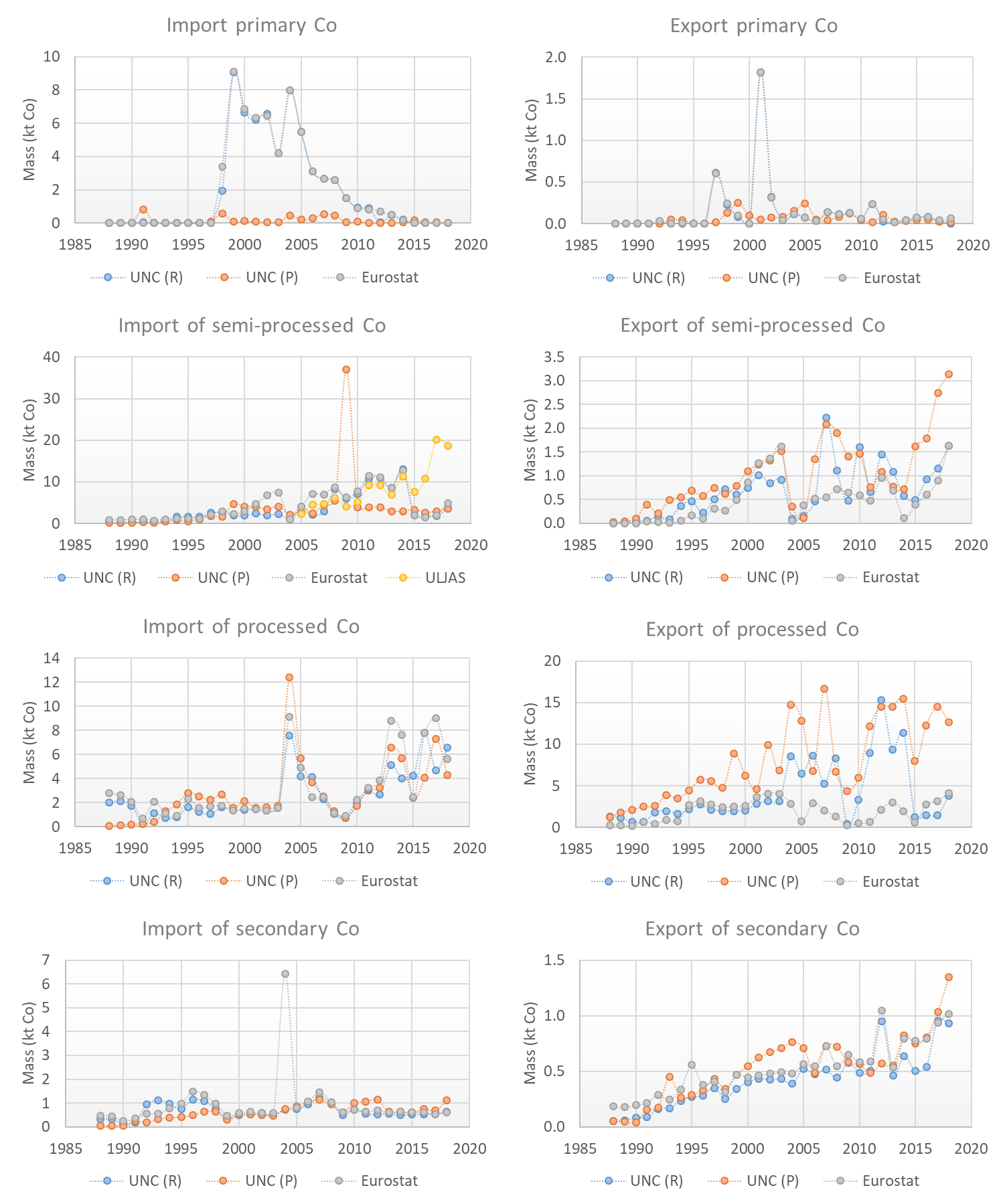


Figure A1. Import in and export from the EU-27 of primary, semi-processed, processed, and secondary Co according to the UN Comtrade (UNC) and Eurostat databases, between 1988 and 2018.. UNC (R): member states as reporters, UNC (P): member states as partners. ULJAS database was also consulted for the import of Co mattes and intermediates to Finland.

# Results per commodity

.
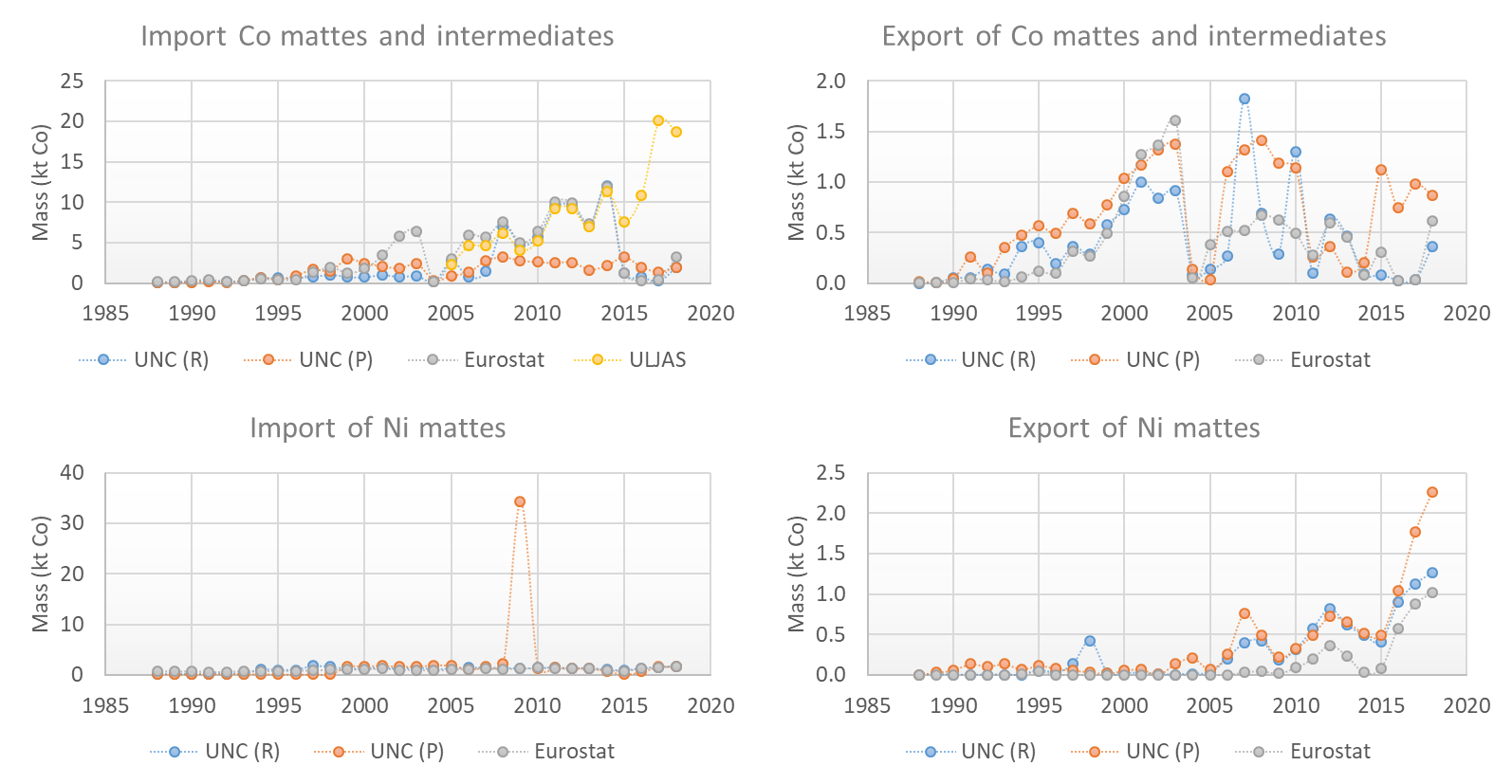


Figure A2. Trade of single commodities of the category semi-processed material according to the UN Comtrade (UNC) and Eurostat databases, between 1988 and 2018 for the EU-27. UNC (R): member states as reporters, UNC (P): member states as partners. ULJAS database was also consulted for the import of Co mattes and intermediates to Finland.


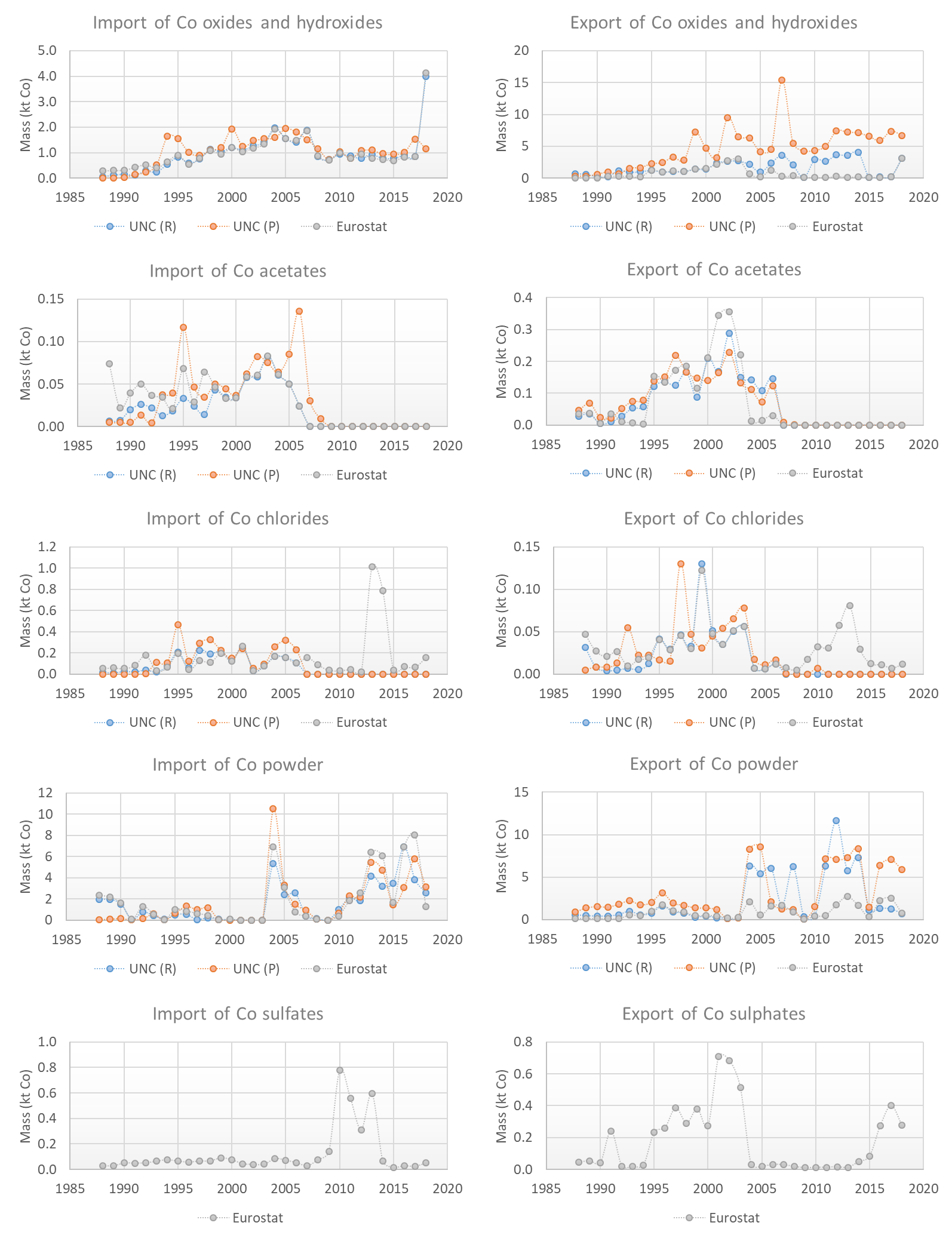


Figure A3. Trade of single commodities of the category processed material according to the UN Comtrade (UNC) and Eurostat databases, between 1988 and 2018 for the EU-27. UNC (R): member states as reporters, UNC (P): member states as partners.


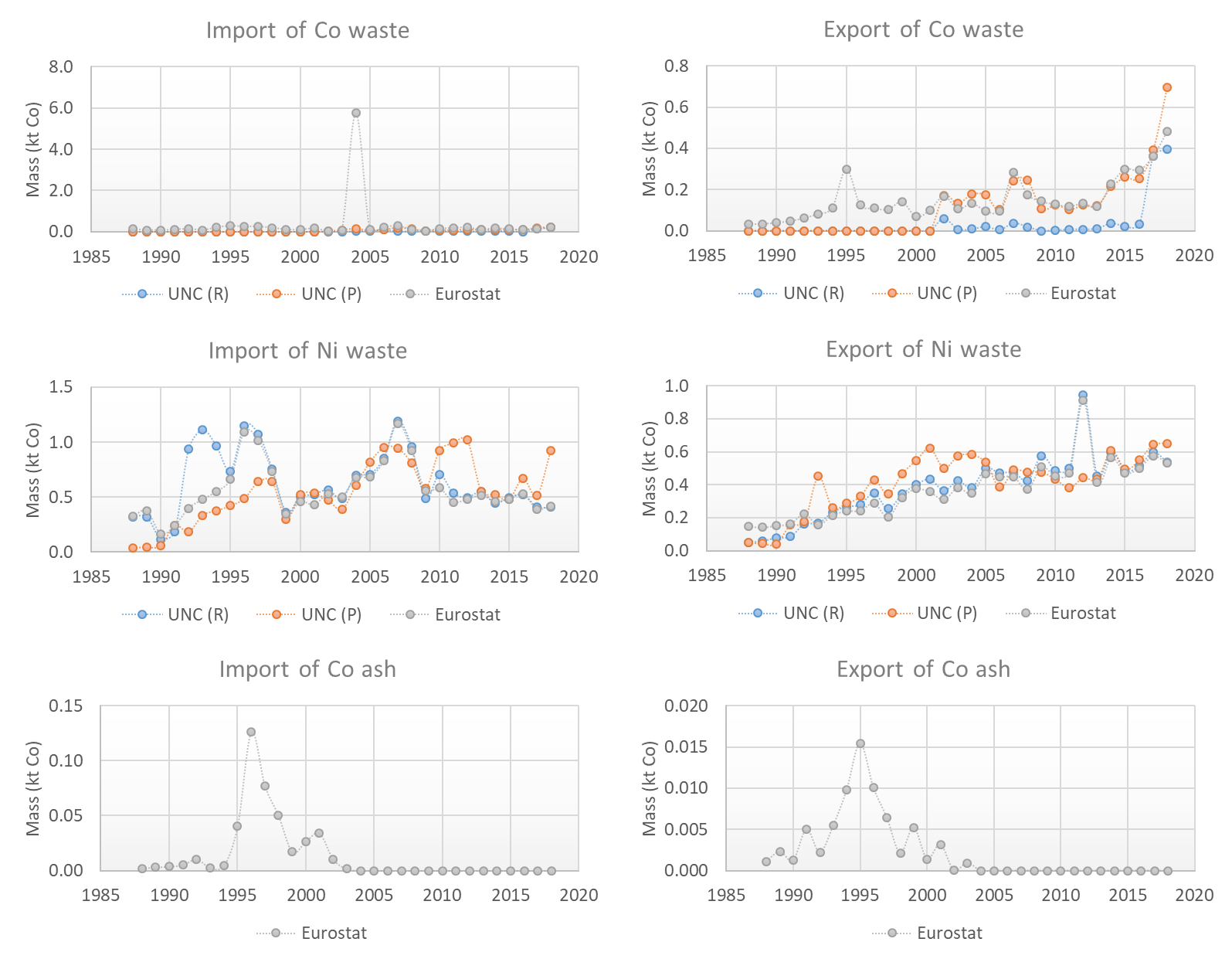


Figure A4. Trade of single commodities of the category secondary material according to the UN Comtrade (UNC) and Eurostat databases, between 1988 and 2018 for the EU-27. UNC (R): member states as reporters, UNC (P): member states as partners

# Results according to main member states


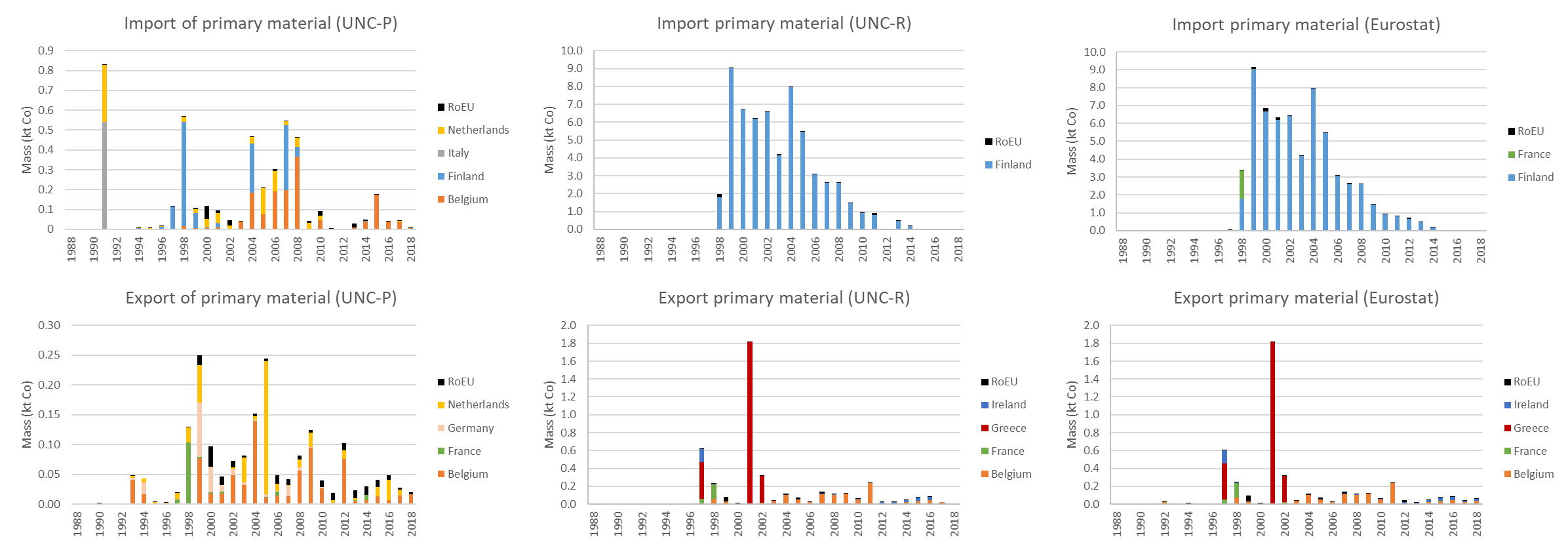


Figure A5. Trade of primary Co for the main involved member states according to the UN Comtrade (UNC) and Eurostat databases, between 1988 and 2018. UNC-R: member states as reporters, UNC-P: member states as partners. RoEU: Rest of EU.

Analysing each member state, the main importers of primary Co have been Belgium and Finland according to UNC as partner, and Finland according to Eurostat and UNC as reporter. For the export, the main member states have been Belgium and the Netherlands according to UNC as partner, and Greece and Belgium according to Eurostat and UNC as reporter.

The countries were selected based on a trade cut-off value of at least 100 tonnes of Co, for at least one year of the studied period. For the import, for Eurostat and UNC-R, the cut-off value was established at 500 tonnes of Co.


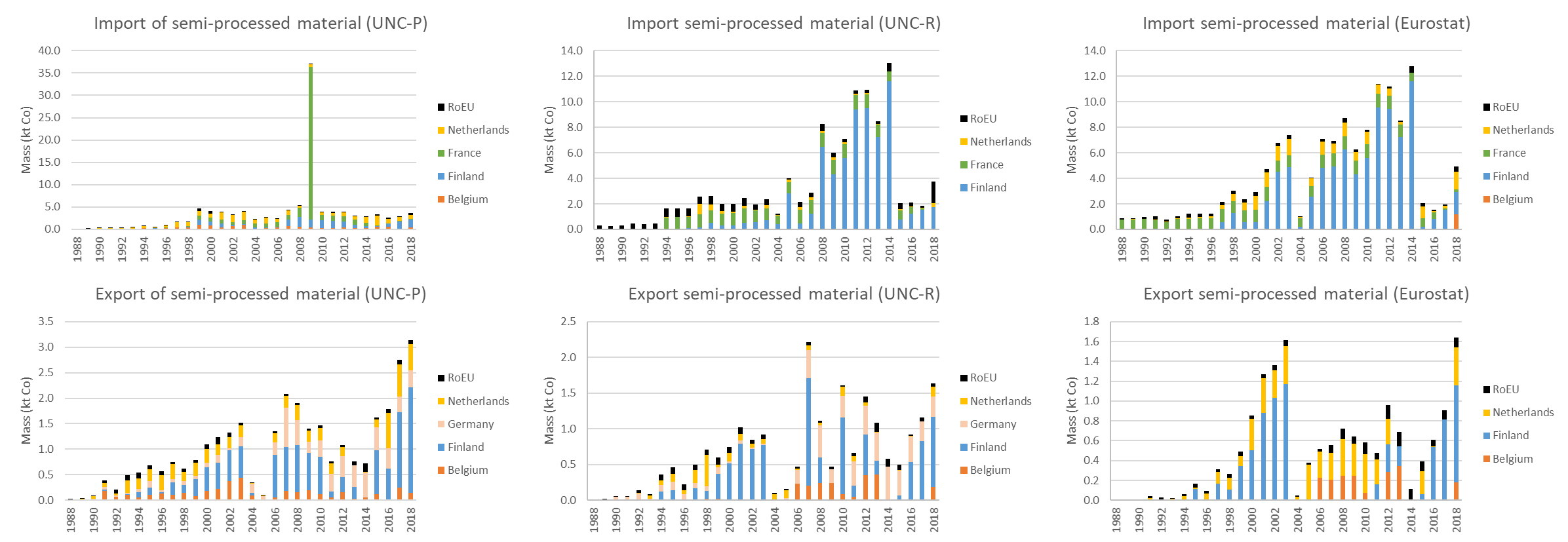


Figure A7. Trade of semi-processed Co for the main involved member states according to the UN Comtrade (UNC) and Eurostat databases, between 1988 and 2018. UNC-R: member states as reporters, UNC-P: member states as partners. RoEU: Rest of EU.

UNC as reporter and as partner report that the main importers of semi-processed Co have been Finland, France, and the Netherlands; and the main exporters Belgium, Finland, Germany, and the Netherlands. Eurostat in turn, reports Finland, France, and the Netherlands as main importers; and Belgium, Czech Republic, France, Germany, the Netherlands, and Sweden as main exporters.

The countries were selected based on a trade cut-off value of at least 500 tonnes of Co, for at least one year of the studied period.


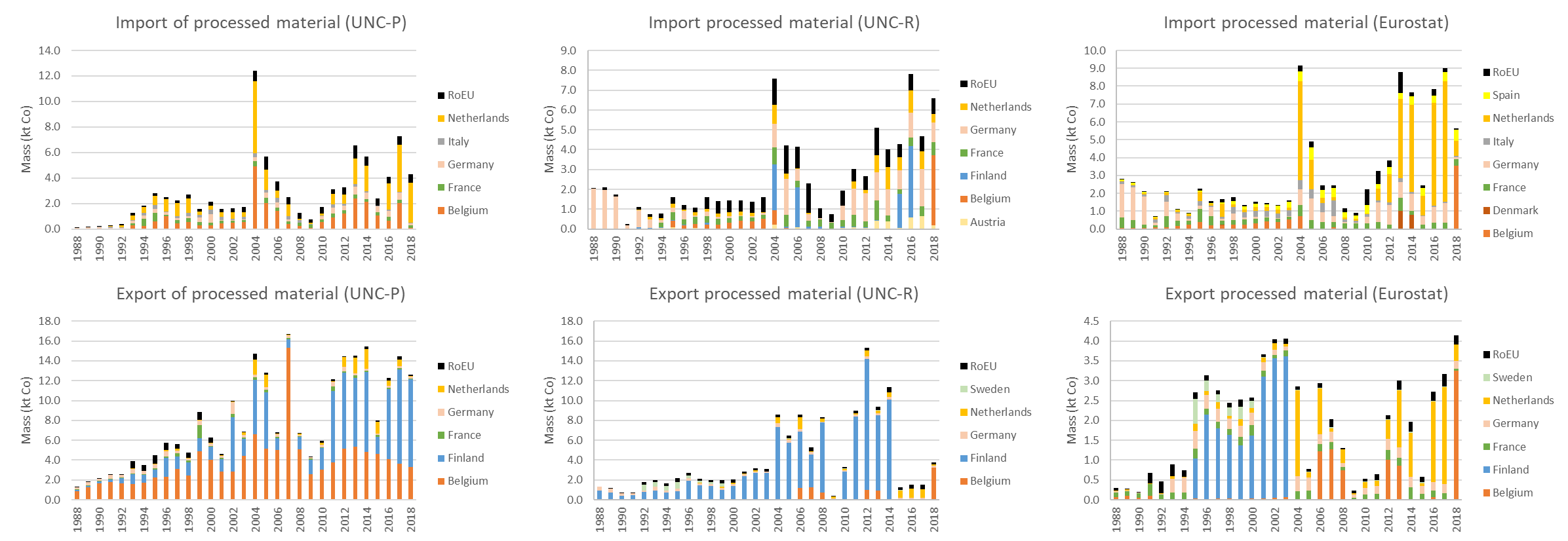


Figure A8. Trade of processed Co for the main involved member states according to the UN Comtrade (UNC) and Eurostat databases, between 1988 and 2018. UNC-R: member states as reporters, UNC-P: member states as partners. RoEU: Rest of EU.

A number of member states have been involved in the trade of these commodities along the studied period. According to the data of UNC as partner, Belgium and Finland have been the main exporters, and Belgium and the Netherlands two of the main importers. According to UNC as reporter, Finland has been the main exporter; for the import, several countries have played a role, such as Belgium, France, and the Netherlands. Eurostat reports that some of the main exporters have been Belgium, Finland, and the Netherlands; and that Belgium, France, and the Netherlands have been some of the main importers.

The countries were selected based on a trade cut-off value of at least 500 tonnes of Co, for at least one year of the studied period.


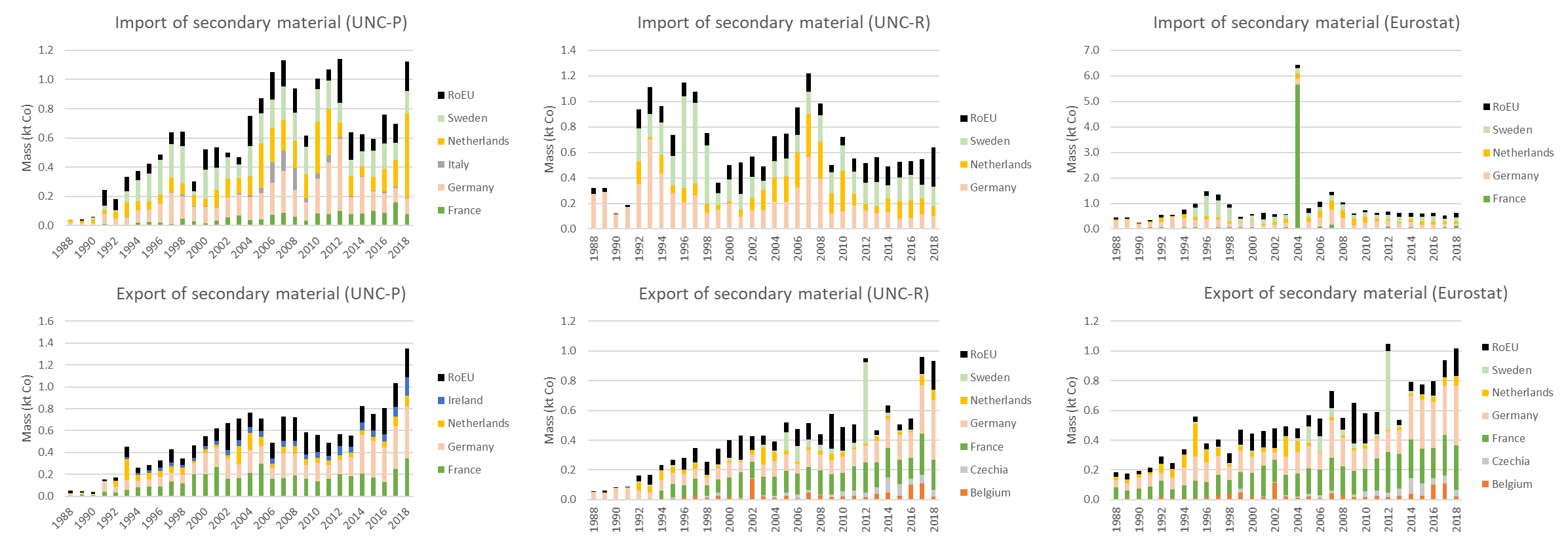


Figure A6. Trade of secondary Co for the main involved member states according to the UN Comtrade (UNC) and Eurostat databases, between 1988 and 2018. UNC-R: member states as reporters, UNC-P: member states as partners. RoEU: Rest of EU.

The countries were selected based on a trade cut-off value of at least 100 tonnes of Co, for at least one year of the studied period.

1. Contribution to the results of the data reported originally in mass, in monetary value, and as special category


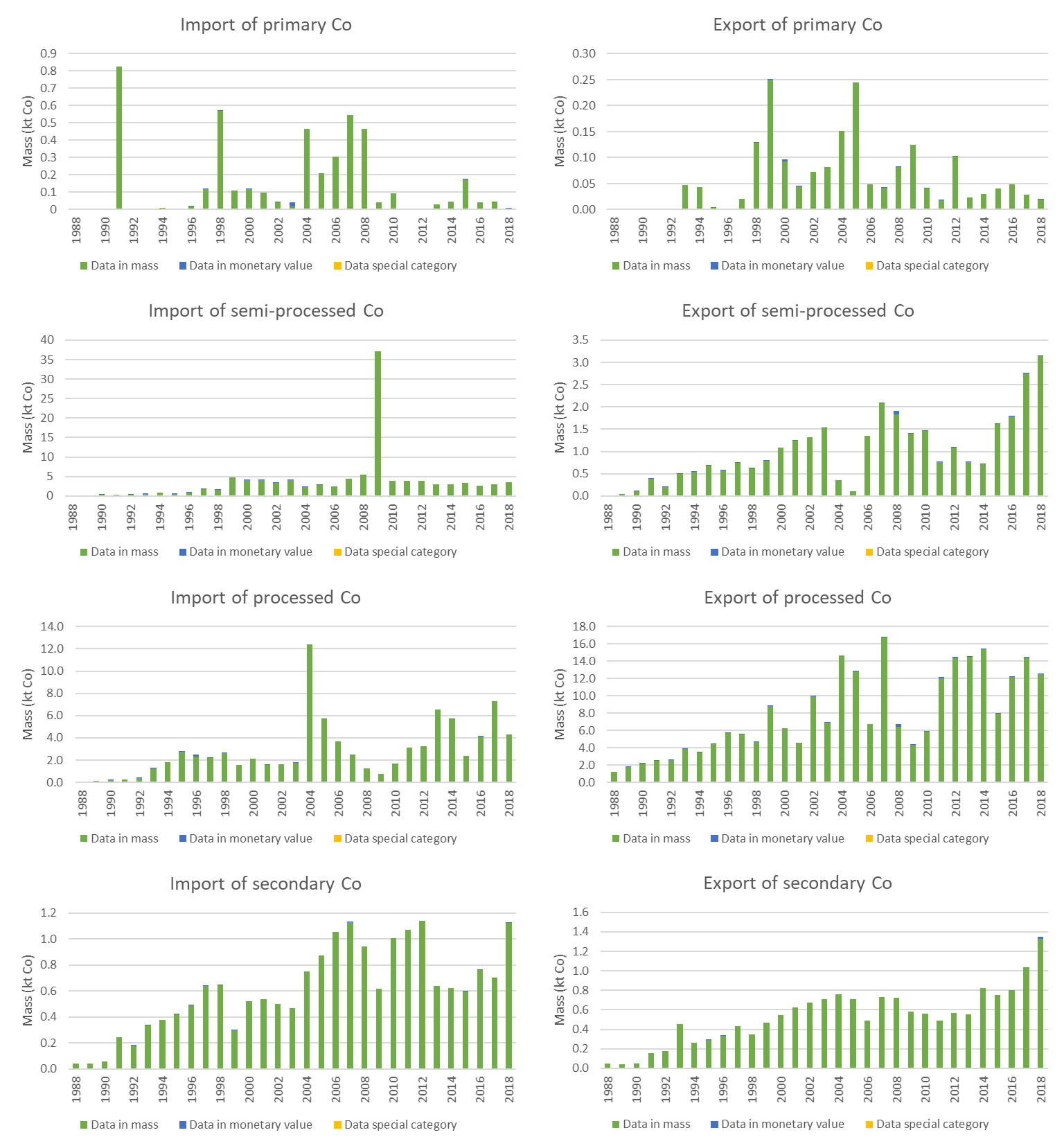


Figure A9. Trade of primary, secondary, semi-processed, and processed Co according to the UN Comtrade database with the member states considered partners, between 1988 and 2018 for the EU-27.


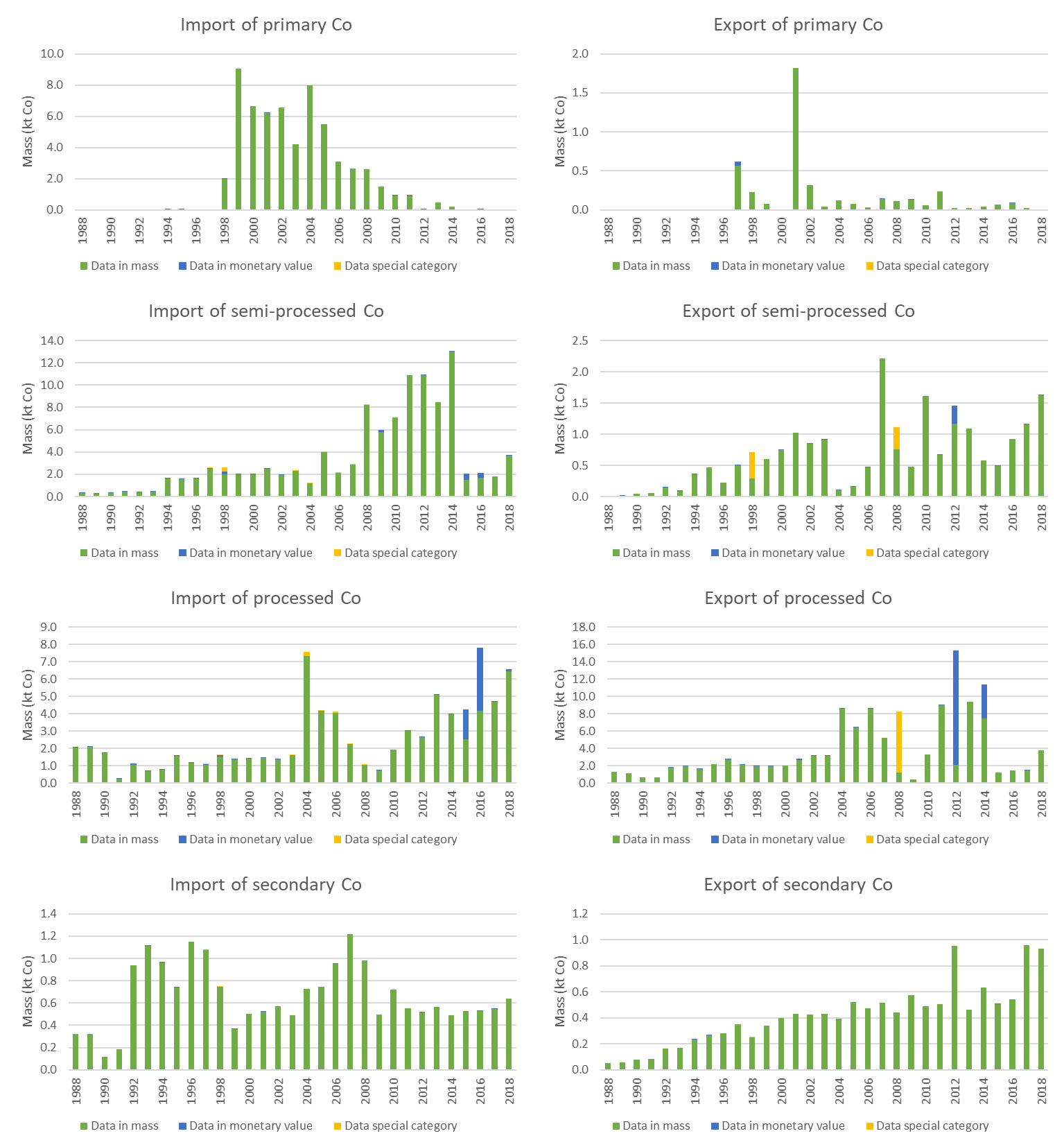


Figure A10. Trade of primary, secondary, semi-processed, and processed Co according to the UN Comtrade database with the member states considered reporters, between 1988 and 2018 for the EU-27.


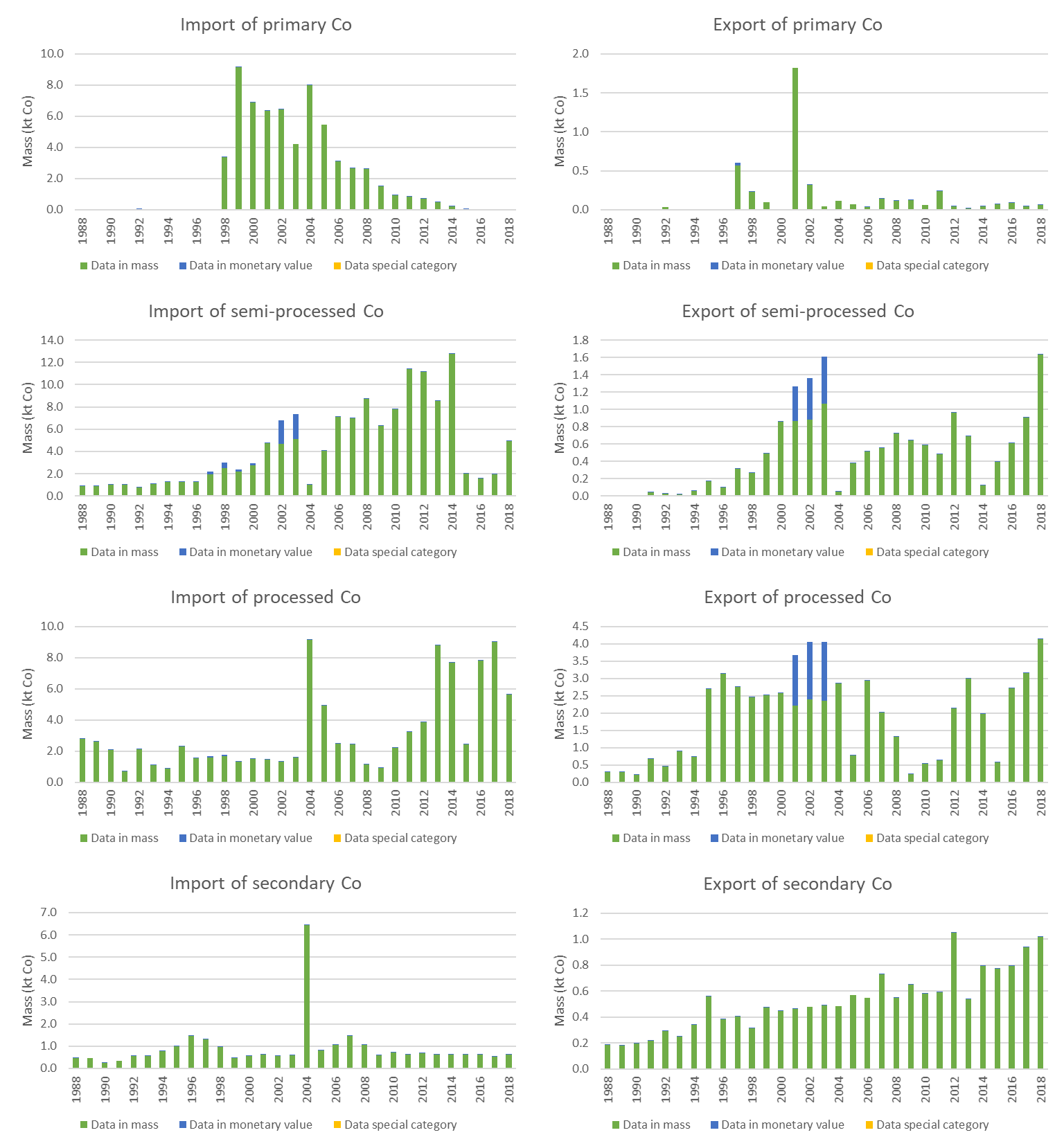


Figure A11. Trade of primary, secondary, semi-processed, and processed Co according to the Eurostat database, between 1988 and 2018 for the EU-27.

# Sensitivity analysis for data originally in monetary value

A sensitivity analysis was performed to analyse the effect of the ratio mass/currency used to transform the data reported in monetary value to mass value. Figure A12, Figure A14, and Figure A16 show the results using the minimum value of the ratio among the different member states. Figure A13, Figure A15, and Figure A17 show the results using the maximum value of the ratio among the different member states. Based on the results showed in section 5, only the results for semi-processed and processed Co are presented.


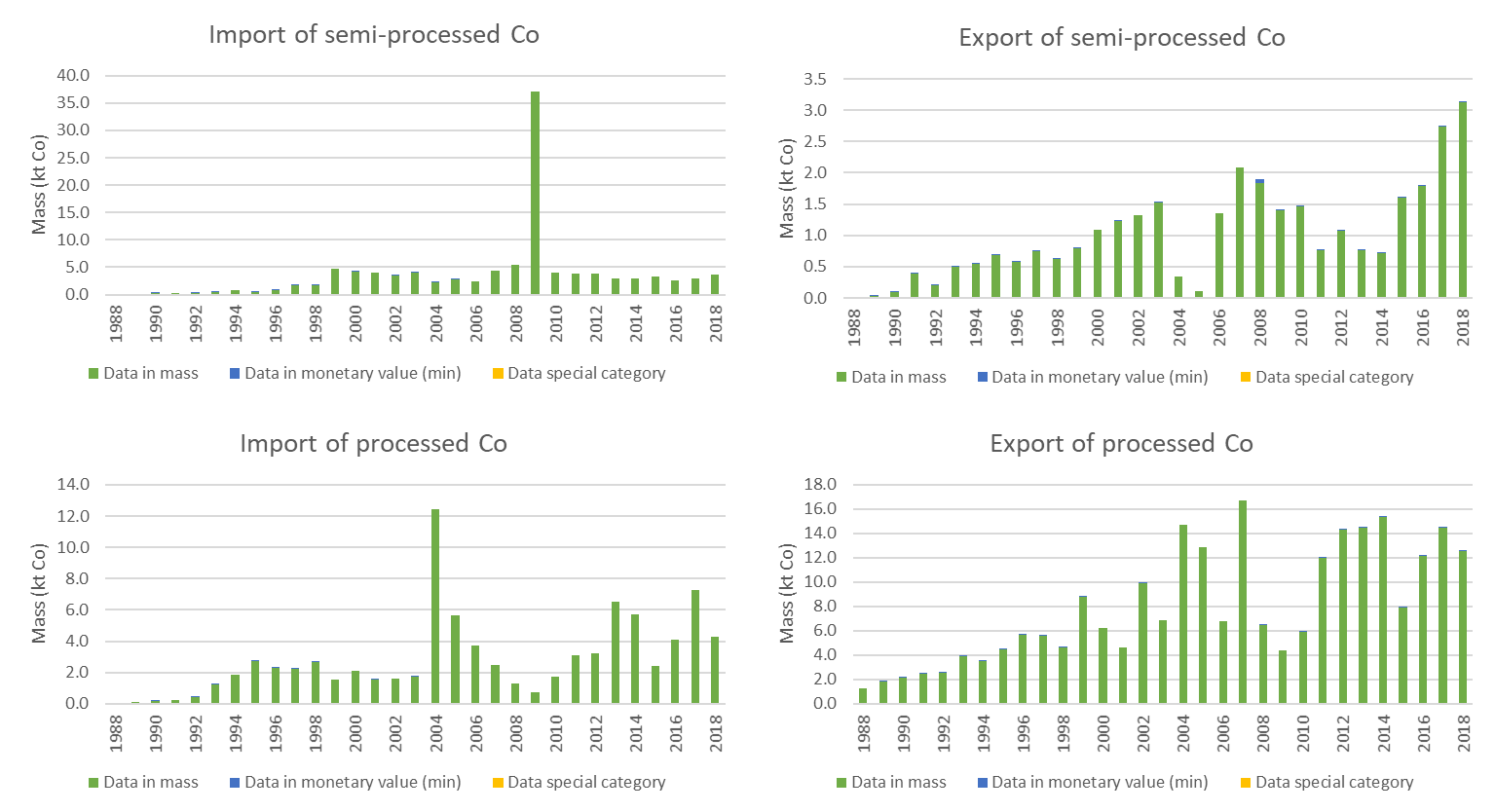


Figure A12. Trade of semi-processed and processed Co according to the UNC database with the member states considered partners, between 1988 and 2018 for the EU-27. The data reported in monetary value was transformed to mass value according to the minimum mass/currency ratio among the member states (per year).


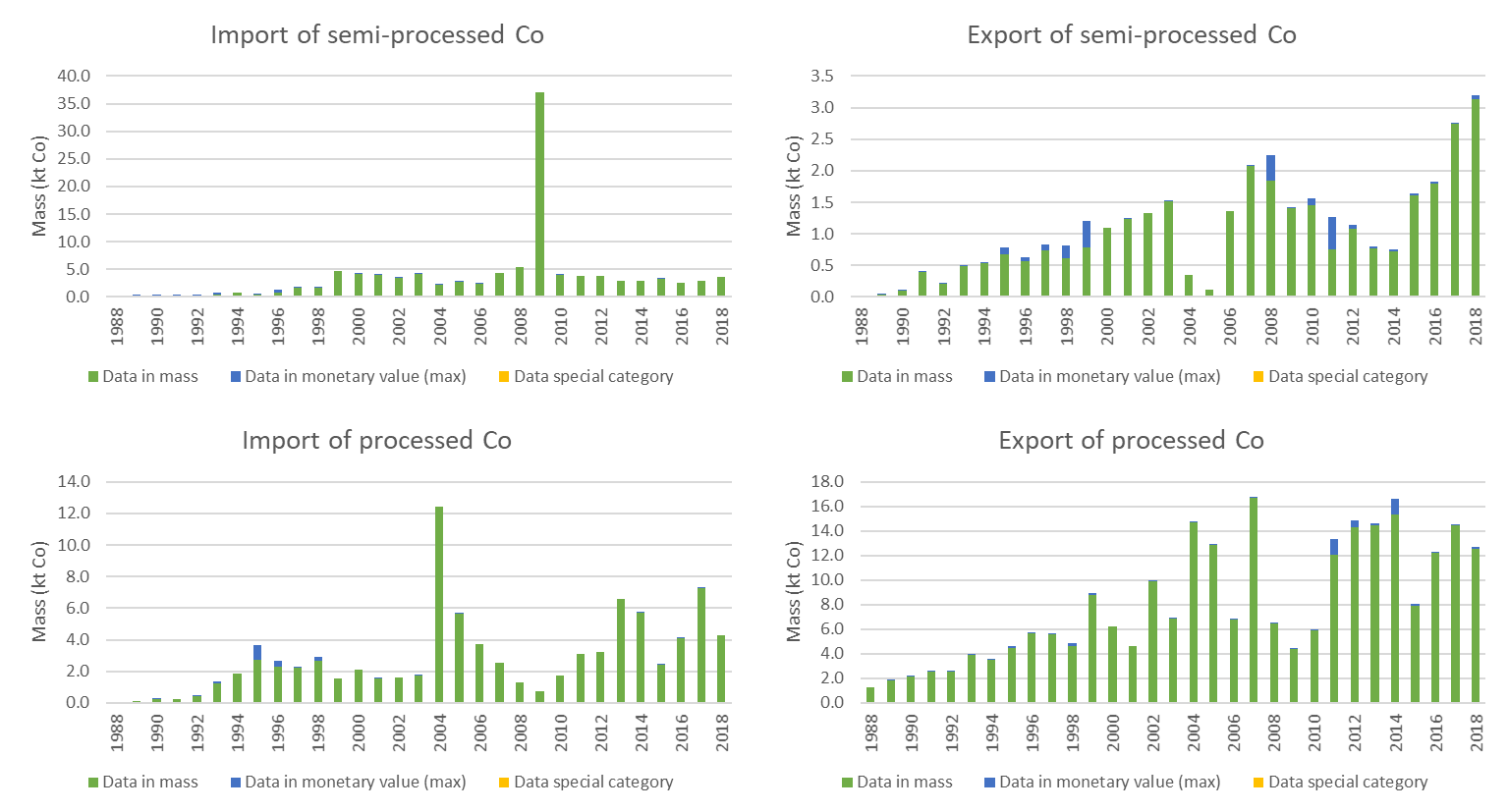


Figure A13. Trade of semi-processed and processed Co according to the UNC database with the member states considered partners, between 1988 and 2018 for the EU-27. The data reported in monetary value was transformed to mass value according to the maximum mass/currency ratio among the member states (per year).


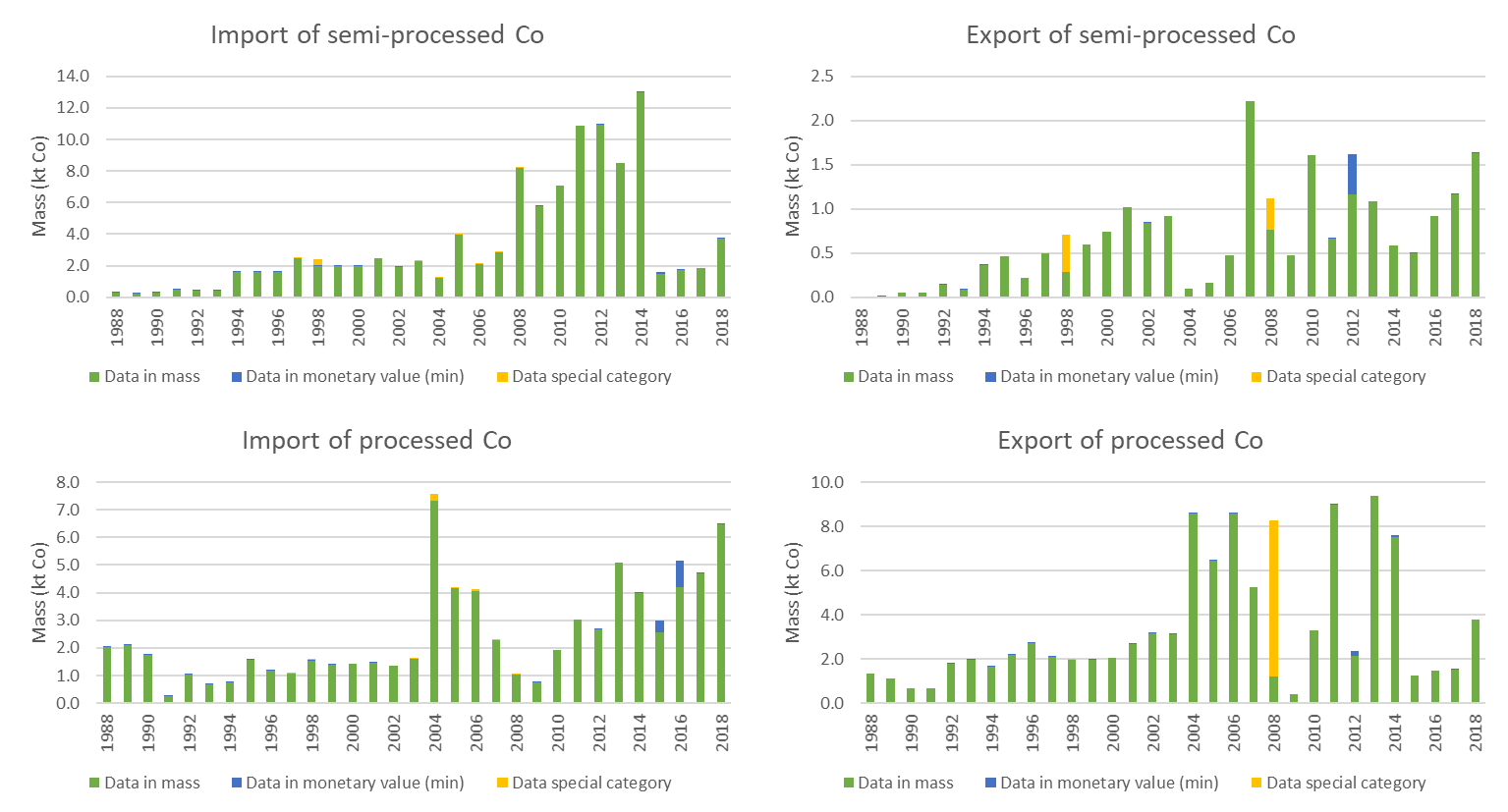


Figure A14. Trade of semi-processed and processed Co according to the UNC database with the member states considered reporters, between 1988 and 2018 for the EU-27. The data reported in monetary value was transformed to mass value according to the minimum mass/currency ratio among the member states (per year).


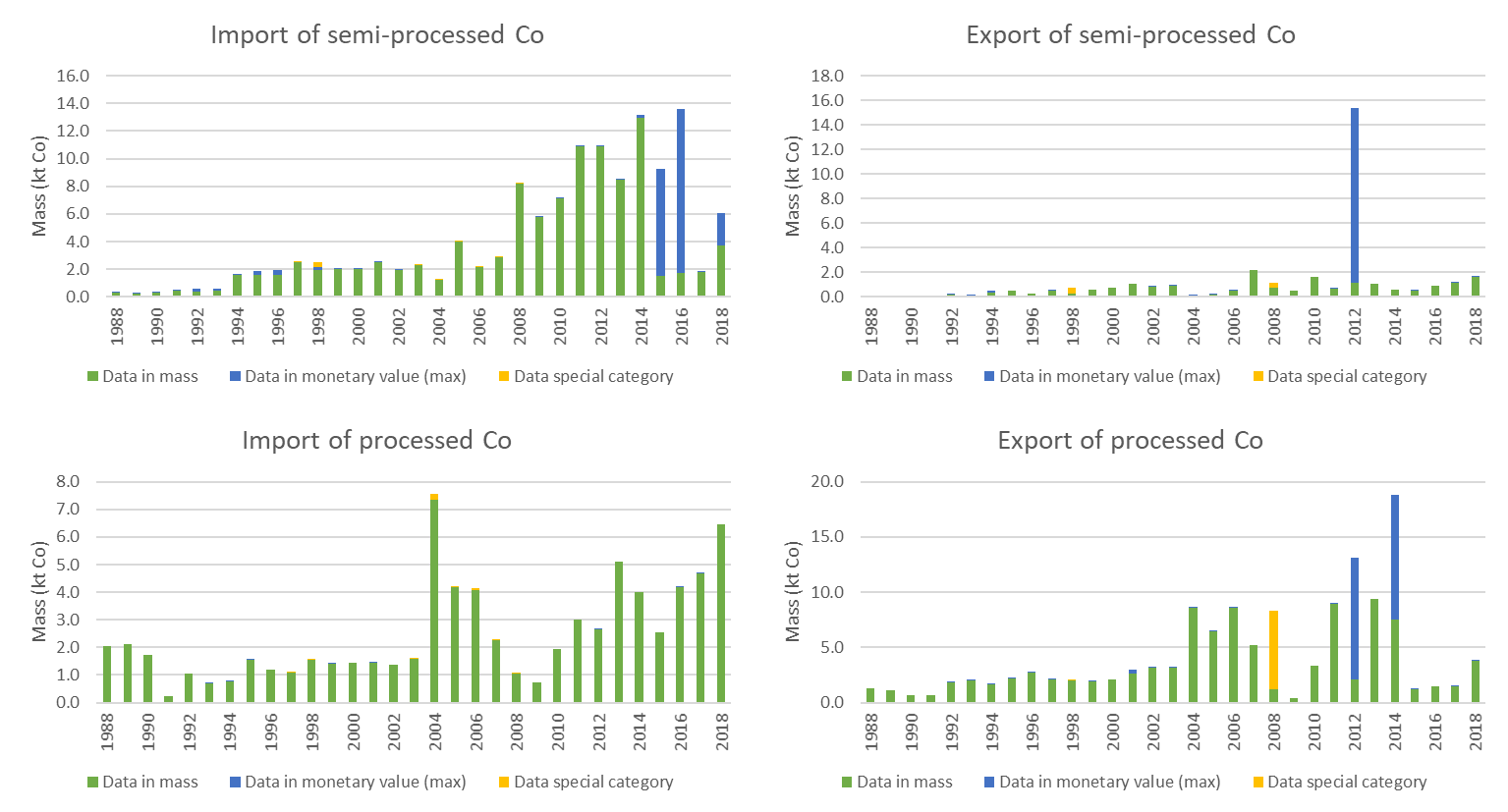


Figure A15. Trade of semi-processed and processed Co according to the UNC database with the member states considered reporters, between 1988 and 2018 for the EU-27. The data reported in monetary value was transformed to mass value according to the maximum mass/currency ratio among the member states (per year).


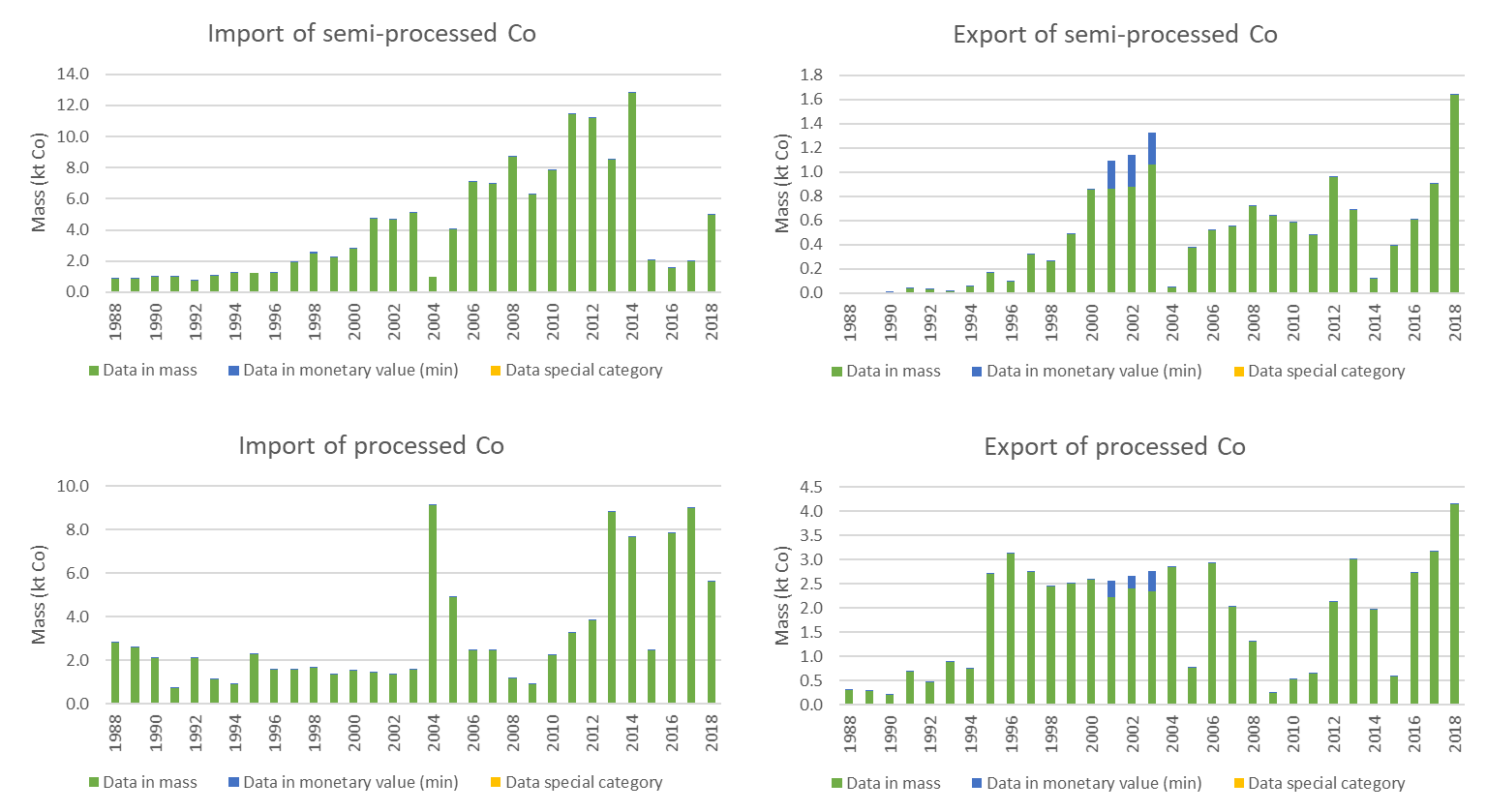


Figure A16. Trade of semi-processed and processed Co according to the Eurostat database, between 1988 and 2018 for the EU-27. The data reported in monetary value was transformed to mass value according to the minimum mass/currency ratio among the member states (per year).


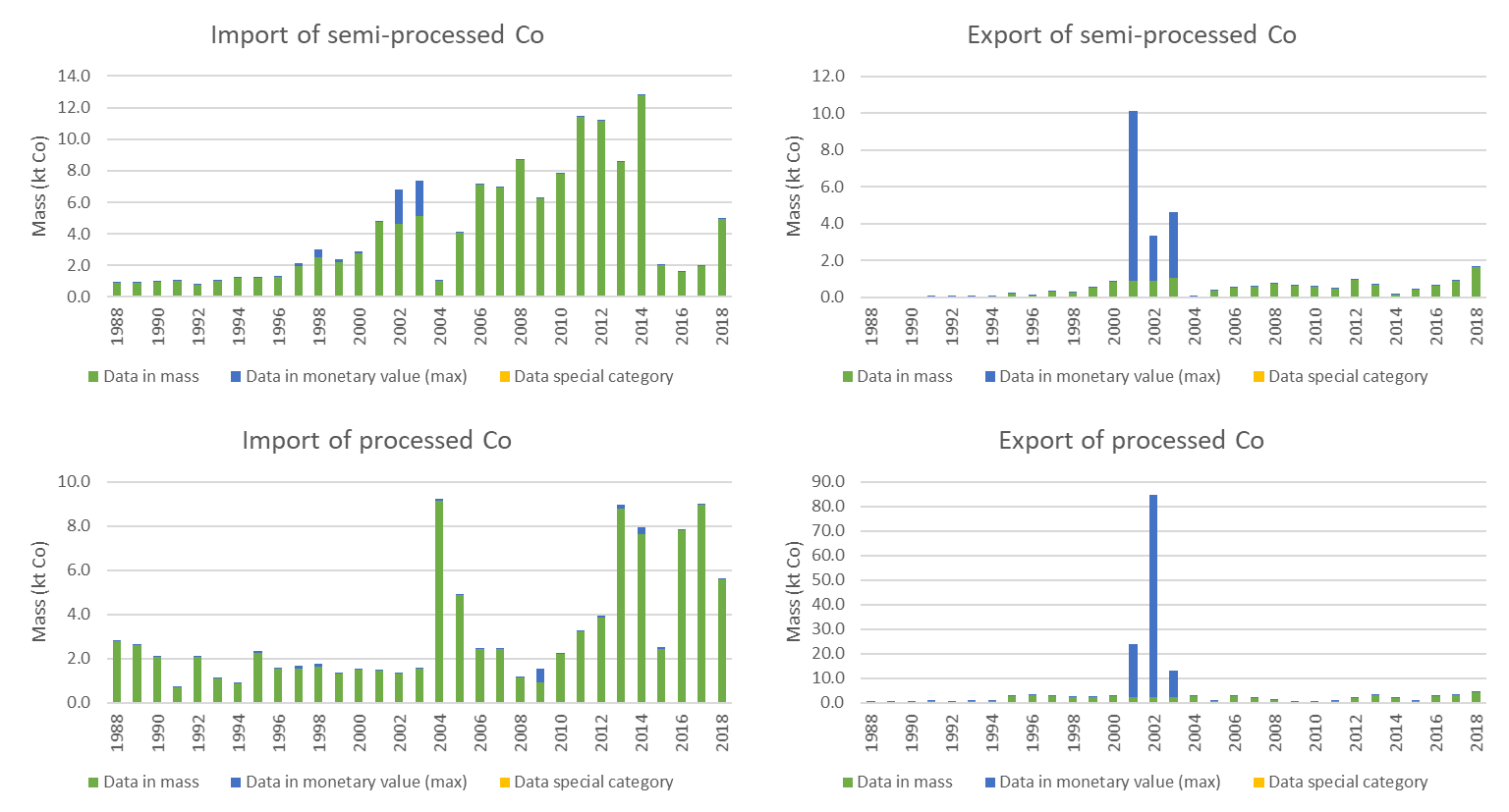


Figure A17. Trade of semi-processed and processed Co according to the Eurostat database, between 1988 and 2018 for the EU-27. The data reported in monetary value was transformed to mass value according to the maximum mass/currency ratio among the member states (per year).

# Sensitivity analysis for sulphate of cobalt

A sensitivity analysis was performed to analyse the effect of the share of sulphates of Co in the commodity “Sulphates of cobalt and of titanium”, recorded in the Eurostat database. Originally, it was assumed 50% share; in the sensitivity analysis a share 25/75 and 75/25 was assumed.


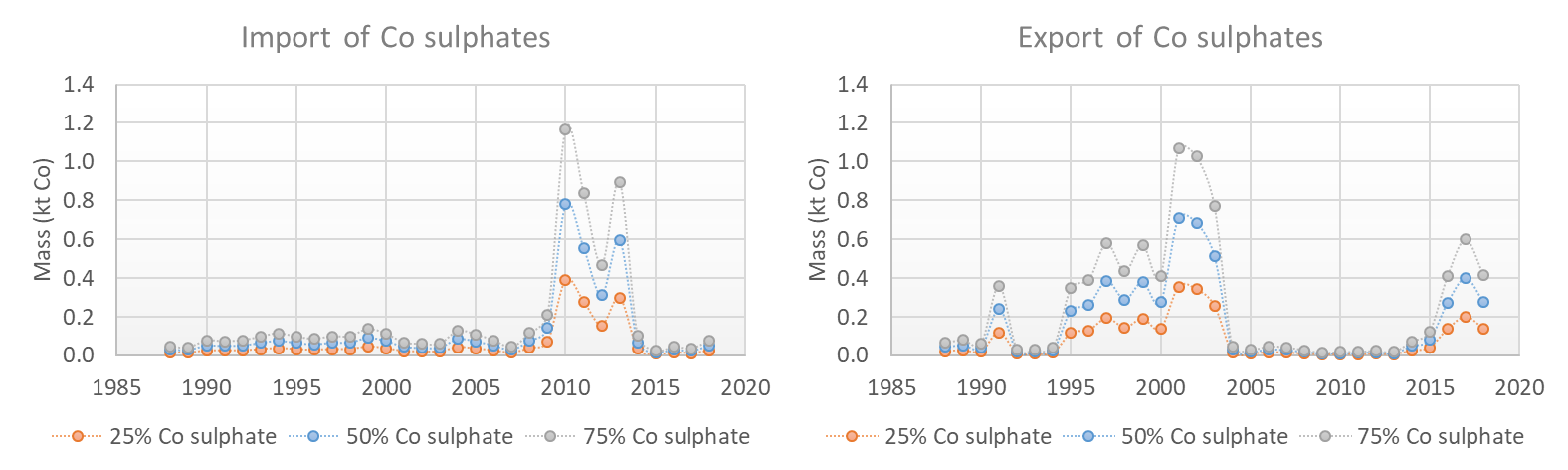


Figure A18. Sensitivity analysis for the trade of Co sulphates, based on different shares of the compound in the commodity “Sulphates of cobalt and of titanium”, according to the Eurostat database between 1988 and 2018 for the EU-27.


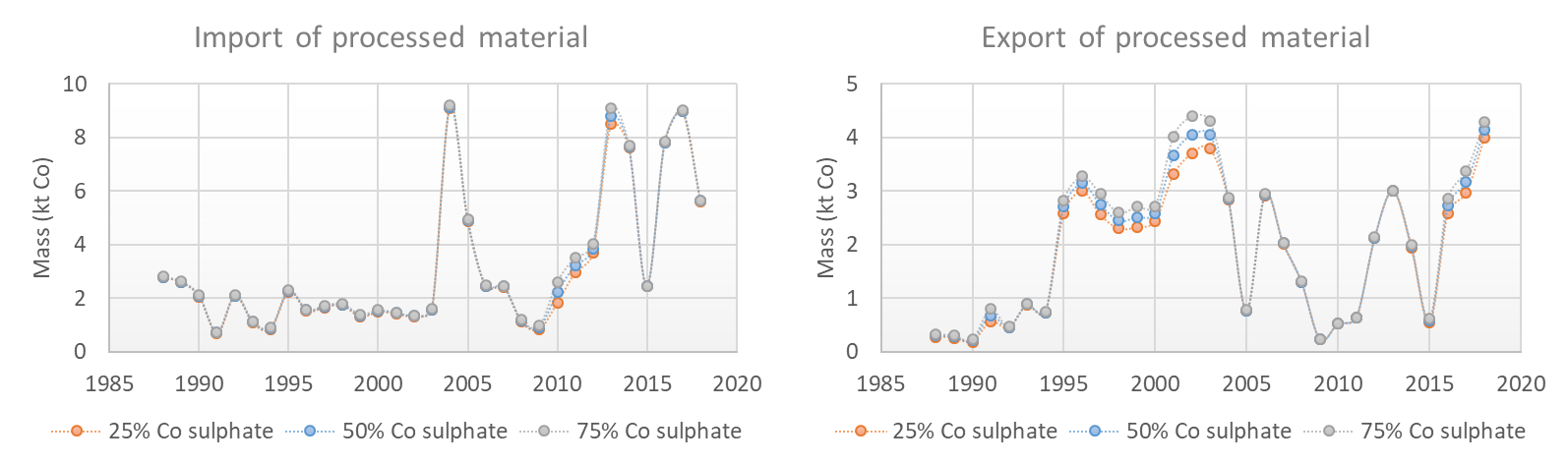


Figure A19. Sensitivity analysis for the trade of processed material, based on different shares of sulphates of Co in the commodity “Sulphates of cobalt and of titanium”, according to the Eurostat database between 1988 and 2018 for the EU-27.

As it is observed in Figure A18, the results present a strong difference between 2010 and 2013 for the import, and in 1991, 1995-2003, and 2016-2018 for the export of Co sulphates. However, in terms of trade of processed material (Figure A19) the effect is negligible, showing for most of the years a difference below 5% in the imports and below 10% in the exports (compared to the base case).
